# Development and Biological Characterization of a Novel Selective TrkA Agonist with Neuroprotective Properties against Amyloid Toxicity

**DOI:** 10.3390/biomedicines10030614

**Published:** 2022-03-06

**Authors:** Thanasis Rogdakis, Despoina Charou, Alessia Latorrata, Eleni Papadimitriou, Alexandros Tsengenes, Christina Athanasiou, Marianna Papadopoulou, Constantina Chalikiopoulou, Theodora Katsila, Isbaal Ramos, Kyriakos C. Prousis, Rebecca C. Wade, Kyriaki Sidiropoulou, Theodora Calogeropoulou, Achille Gravanis, Ioannis Charalampopoulos

**Affiliations:** 1 Department of Pharmacology, Medical School, University of Crete, 71003 Heraklion, Greece; thanasis_rogdakis@imbb.forth.gr (T.R.); despoina_charou@imbb.forth.gr (D.C.); elenipapadimitriou95@yahoo.gr (E.P.); medp2011866@med.uoc.gr (M.P.); gravania@uoc.gr (A.G.); 2 Foundation for Research & Technology-Hellas (IMBB-FORTH), Institute of Molecular Biology & Biotechnology, 70013 Heraklion, Greece; sidirop@uoc.gr; 3 National Hellenic Research Foundation, Institute of Chemical Biology, 11635 Athens, Greece; alatorrata@eie.gr (A.L.); cchalikio@eie.gr (C.C.); thkatsila@eie.gr (T.K.); kyrprous@eie.gr (K.C.P.); tcalog@eie.gr (T.C.); 4 Molecular and Cellular Modeling Group, Heidelberg Institute for Theoretical Studies (HITS), 69118 Heidelberg, Germany; alexandros.tsengenes@h-its.org (A.T.); christina.athanasiou@h-its.org (C.A.); rebecca.wade@h-its.org (R.C.W.); 5 Faculty of Biosciences, Heidelberg University, 69120 Heidelberg, Germany; 6 Heidelberg Biosciences International Graduate School, Heidelberg University, 69120 Heidelberg, Germany; 7 Innovative Technologies in Biological Systems SL (INNOPROT), 48160 Bizkaia, Spain; iramos@innoprot.com; 8 Center for Molecular Biology (ZMBH), DKFZ-ZMBH Alliance, Heidelberg University, 69120 Heidelberg, Germany; 9 Interdisciplinary Center for Scientific Computing (IWR), Heidelberg University, 69120 Heidelberg, Germany; 10 Department of Biology, University of Crete, 71113 Heraklion, Greece

**Keywords:** neurotrophin, TrkA neurotrophin receptor, nerve growth factor, Alzheimer disease, amyloid-beta, neurotrophin mimetic, neuronal survival, synapse, LTP

## Abstract

Neurotrophins are growth factors that exert important neuroprotective effects by preventing neuronal death and synaptic loss. Nerve Growth Factor (NGF) acts through the activation of its high-affinity, pro-survival TrkA and low-affinity, pro-apoptotic p75^NTR^ receptors. NGF has been shown to slow or prevent neurodegenerative signals in Alzheimer’s Disease (AD) progression. However, its low bioavailability and its blood–brain-barrier impermeability limit the use of NGF as a potential therapeutic agent against AD. Based on our previous findings on synthetic dehydroepiandrosterone derivatives, we identified a novel NGF mimetic, named ENT-A013, which selectively activates TrkA and exerts neuroprotective, anti-amyloid-β actions. We now report the chemical synthesis, in silico modelling, metabolic stability, CYP-mediated reaction phenotyping and biological characterization of ENT-A013 under physiological and neurodegenerative conditions. We show that ENT-A013 selectively activates the TrkA receptor and its downstream kinases Akt and Erk1/2 in PC12 cells, protecting these cells from serum deprivation-induced cell death. Moreover, ENT-A013 promotes survival of primary Dorsal Root Ganglion (DRG) neurons upon NGF withdrawal and protects hippocampal neurons against Amyloid β-induced apoptosis and synaptic loss. Furthermore, this neurotrophin mimetic partially restores LTP impairment. In conclusion, ENT-A013 represents a promising new lead molecule for developing therapeutics against neurodegenerative disorders, such as Alzheimer’s Disease, selectively targeting TrkA-mediated pro-survival signals.

## 1. Introduction

Endogenous neurotrophic factors constitute key regulators of neuronal survival and repair during development but most importantly during adulthood and aging. Nerve Growth Factor (NGF) was the first neurotrophin discovered and its activity is crucial for neuronal development, growth and survival, as well as synaptic functioning in the adult brain. NGF exerts its neurogenic and neuroprotective effects by acting through its specific and high-affinity TrkA receptor. Upon NGF binding to TrkA and autophosphorylation of the latter, a cascade of signaling occurs, including Akt and Erk1/2 activation, ultimately leading to the transcriptional regulation of anti-apoptotic proteins such as Bcl2 [1]. There is a large amount of evidence indicating that neurotrophin cellular processing and expression levels are deregulated in neurodegenerative conditions like Alzheimer’s Disease (AD), and this has been postulated to significantly contribute to the disease’s pathology [2].

AD is a devastating neurodegenerative disorder, which currently affects more than 50 million people worldwide. Clinically, the disease is characterized by severe memory loss and cognitive impairment, leaving patients with difficulties in performing simple everyday tasks [3]. Pathologically, the hallmarks of the disease include the deposition of amyloid-β plaques extracellularly and the presence of hyperphosphorylated neurofibrillary tau tangles intracellularly. It is well accepted that the elevated levels of misprocessed Aβ oligomers, which then form the Aβ plaques, are the initiating step in the AD pathology [4]. This elevation is partly responsible for increased astrogliosis, decreased neurogenic potential, impaired synaptic functioning and, ultimately, neuronal death. A population of neurons that is severely affected in the early stages of AD is the cholinergic neurons [5,6]. More specifically, it is well documented that BFCN express both NGF receptors, as well as being capable of producing and secreting NGF [7,8]. NGF signaling is critical for the survival and proper functioning of these affected neurons, while its depletion in a mouse model resulted in AD-like pathology, including Aβ plaques formation, tau hyperphosphorylation and synaptic dysfunction [9]. Furthermore, NGF treatment has been shown to ameliorate the Aβ-induced phenotype, preventing memory impairment [10].

However, the therapeutic use of NGF is hindered by its biochemical and biological properties. It has limited ability to cross the blood–brain barrier and poor pharmacokinetic properties, making it unstable in serum. Additionally, it is vulnerable to proteolytic cleavage and it cannot diffuse in the CNS parenchyma. Nevertheless, there have been several attempts to bypass these limitations by delivering NGF via intracerebroventricular infusion or via gene therapy using an AAV2 viral vector [11,12,13]. Both these clinical trials were unsuccessful, mainly due to NGF-mediated undesirable hyperalgesic secondary effects. This could be attributed to the late non-reversible stage of the disease or to the restricted virus diffusion limiting the number of neurons that were affected by NGF [14].

An alternative pharmacological approach is the design and development of small-sized molecules that could mimic NGF activity and promote neuronal survival and functioning. Our research team has previously shown that DHEA, an endogenous neurosteroid produced by neurons and glia in the Central Nervous System (CNS), has the ability to bind to and activate both NGF receptors, namely TrkA and p75^NTR^, with high affinity, resulting in anti-apoptotic effects [15]. Indeed, BNN27, a C-17-spiro derivative of DHEA, has been previously developed, lacking the estrogenic or androgenic activity of the parent molecule, DHEA, but sustaining neuroprotective, anti-inflammatory and neurogenic properties, acting also through TrkA and p75^NTR^ receptors [16,17]. Interestingly, administration of BNN27 in 5 × FAD animals (experimental mouse model for AD) resulted in improved performance in the Y-maze memory task, protection against synapse degeneration, reduced astrogliosis and increased neurogenesis (manuscript in preparation).

BNN27 serves as a lead molecule to synthesize and biologically test novel DHEA derivatives that are selective agonists of the TrkA receptor, thereby exerting neuroprotective effects and thus having the potential to be used as drugs against neurodegenerative diseases such as AD. In the present study, we present ENT-A013, a novel and selective TrkA agonist, which mimics NGF by activating TrkA and its downstream signaling kinases Akt and Erk1/2. ENT-A013 is a chemically stable and more potent compound compared to BNN27, while it targets only the pro-survival TrkA receptor without any pro-apoptotic p75^NTR^-mediated effects. We describe its chemical synthesis, potential binding sites on the extracellular part of the TrkA receptor, its metabolic stability and CYP-mediated reaction phenotyping. In addition, biological evaluation of ENT-A013 was performed, revealing that it exerts outstanding neuroprotective abilities in PC12 cells and primary Dorsal Root Ganglia neurons challenged with serum deprivation and NGF deprivation, respectively. Furthermore, we report that ENT-A013 can positively modulate APP processing while it protects primary hippocampal neurons against oligomeric Aβ toxicity. Finally, ENT-A013 enhances synaptic functioning by reversing Aβ-induced synapse degeneration and LTP impairment. Hence, we propose ENT-A013 as a lead molecule for the development of selective TrkA-mediated neuroprotective agents for the pharmacological management of AD.

## 2. Materials and Methods

### 2.1. Synthesis of ENT-A013

Reactions were run in flame-dried glassware under an atmosphere of argon or nitrogen. All solvents were dried and/or purified according to standard procedures prior to use. Melting points were determined with an Electrothermal Digital Melting Point Apparatus, Cole-Parmer ET0001/Version 1.0, and were uncorrected. Optical rotations were measured using a P3000 series polarimeter (Krüss Optronic GmbH, Hamburg, Germany). NMR spectra were recorded on Varian spectrometers (Varian Medical Systems, Inc., Palo Alto, CA, USA). ^1^H NMR spectra were recorded at 300 MHz or 600 MHz; ^13^C NMR spectra were recorded at 75 MHz or 150 MHz and were internally referenced to residual solvent peaks. Chemical shifts are reported in *δ* units, parts per million (ppm). Low resolution mass spectra were recorded on an LC-MSn Fleet mass spectrometer (Thermo Fisher Scientific, Waltham, MA, USA) using MeOH as the solvent. High resolution mass spectrometry (HRMS) spectra were recorded on a UPLC-MSn Orbitrap Velos mass spectrometer (Thermo Fisher Scientific, Waltham, MA, USA). Flash column chromatography (FCC) was performed on silica gel 60 (230–400 mesh, Merck KGaA, Darmstadt, Germany) and thin layer chromatography (TLC) on precoated glass plates 60 F254 (0.2 mm, Merck KGaA, Darmstadt, Germany). Spots were visualized using UV light at 254 nm and phosphomolybdic acid stain (PMA, 10% in absolute ethanol).

### 2.2. Synthesis of (E)-(3β-Hydroxy-5-Androsten-17-Ylidene)Ethyl Ester (1)

To a solution of DHEA (2.0 g, 6.94 mmol) and triethyl phosphonoacetate (15.46 mL, 77.9 mmol) in anhydrous tetrahydrofuran (THF)/absolute ethanol 1:1 (21.4/21.4 mL) was added dropwise at room temperature (RT) a solution of sodium ethoxide (EtONa) in absolute ethanol (prepared from 1.59 g Na in 30 mL ethanol). The resulting mixture was refluxed overnight. Subsequently, the reaction was cooled to 0 °C and was carefully quenched with water and acidified using 10% aq. HCl until completion of formation of a precipitate. The solid was filtered under reduced pressure, washed with water (30 mL × 3 times) and petroleum ether 40–60 °C (30 mL × 3 times), and air-dried. The solid was dissolved in CH_2_Cl_2_ and the solution was dried over anhydrous Na_2_SO_4_ and filtered. The filtrate was then evaporated under reduced pressure to afford compound 1 (2.39 g, 96% yield) as a pure white crystalline solid. The compound was used in the next step without further purification. Mp: 178–180 °C; ${\left[a\right]}_{D}^{25}$ = −78° (c = 0.006 g/mL, CHCl_3_); R_F_: 0.5 (petroleum ether 40–60 °C/acetone 80:20); ^1^H NMR (600 MHz, CDCl_3_): *δ* 0.84 (s, 3H), 1.03 (s, 3H), 1.28 (t, *J* = 7.1 Hz, 3H, OCH_2_*CH_3_*), 1.31–2.34 (m, 18H), 2.78–2.90 (m, 2H), 3.48–3.57 (m, 1H, 3*α*-H), 4.15 (q, *J* = 7.1 Hz, 2H, O*CH_2_*CH_3_), 5.36 (d, *J* = 5.0 Hz, 1H, 6-H), 5.55 (bs, 1H, 20-H); ^13^C NMR (150 MHz, CDCl_3_): *δ* 14.5, 18.4, 19.6, 21.1, 24.6, 30.6, 31.7, 31.8, 35.3, 36.8, 37.4, 42.4, 46.2, 50.4, 54.0, 59.7, 71.7, 71.8, 108.8, 121.5, 140.9, 167.6, 176.3; ESI-HRMS: *m*/*z* [M + H]^+^ calcd. for C_23_H_35_O_3_ 359.2580, found 359.2581; *m*/*z* [M + Na]^+^ calcd. for C_23_H_34_O_3_Na [M + Na]^+^ 381.2399, found 381.2400.

### 2.3. Synthesis of (E)-[3β-(t-Butyldimethylsilyloxy)-5-Androsten-17-Ylidene]Ethyl Ester (2)

To a solution of compound 1 (2.25 g, 6.28 mmol) in anhydrous THF (20 mL) imidazole (1.33 g, 19.5 mmol) and iodine (4.76 g, 37.5 mmol) were added at 0 °C and the mixture was stirred at 0 °C for 30 min. Subsequently, *tert*-butyldimethylsilyl chloride (1.05 g, 6.67 mmol) was added and the resulting mixture was stirred at 25 °C overnight. After completion of the reaction, the solvent was evaporated in vacuo and the residue was diluted and extracted using ethyl acetate (EtOAc). The organic layer was washed using sat. aq. Na_2_S_2_O_4_, brine and dried over anhydrous Na_2_SO_4_. The solvent was removed in vacuo and the residue was purified by FCC (elution solvent: petroleum ether 40–60 °C/ethyl acetate 95:5) to afford compound 2 as a white solid (2.36 g, 93% yield). Mp: 100–102 °C; ${\left[a\right]}_{D}^{25}$ = −50° (c = 0.0056 g/mL, CHCl_3_); R_F_: 0.86 (petroleum ether 40–60 °C/EtOAc 80:20); ^1^H NMR (600 MHz, CDCl_3_): *δ* 0.05 (s, 6H, Si(CH_3_)_2_), 0.83 (s, 3H), 0.89 (s, 9H, C(CH_3_)_3_), 1.02 (s, 3H), 1.28 (t, *J* = 7.1 Hz, 3H, OCH_2_*CH_3_*), 1.32–2.31 (m, 17H), 2.79–2.89 (m, 2H) 3.42–3.54 (m, 1H, 3*α*-H), 4.15 (q, *J*= 7.1 Hz, 2H, O*CH_2_*CH_3_), 5.33 (d, *J* = 5.3 Hz, 1H, 6-H), 5.54 (t, *J* = 2.3 Hz, 1H, 20-H); ^13^C NMR (75 MHz, CDCl_3_): *δ* −4.3, 14.5, 18.4, 19.6, 21.1, 24.6, 26.1, 30.6, 31.7, 31.8, 32.2, 35.4, 36.8, 37.5, 42.9, 46.2, 50.5, 54.0, 59.7, 72.7, 108.8, 120.9, 121.0, 141.8, 167.6, 176.4; ESI-HRMS: *m*/*z* [M + H]^+^ calcd. for C_29_H_49_O_3_Si 473.3445, found 473.3445; *m*/*z* [M + Na]^+^ calcd. for C_29_H_48_O_3_SiNa [M + Na]^+^ 495.3263, found 495.3265.

### 2.4. Synthesis of (E)-3β-(t-Butyldimethylsilyloxy)-Pregna-5,17(20)-Dien-21-ol (3)

To a solution of compound 2 (2.20 g, 4.65 mmol) in anhydrous ΤHF (94 mL) was added dropwise at −78 °C diisobutylaluminum hydride (DIBAL-H) [(1.0 M in hexane), 18.7 mL, 18.7 mmol]. The reaction mixture was stirred at −78 °C for 2.5 h and at 25 °C for an additional 1 h. After completion of the reaction, saturated aqueous NH_4_Cl (40 mL) was added at 0 °C and the solvent was evaporated in vacuo. EtOAc (30 mL) was added to the residue and the organic layer was washed using 10% aq. HCl (20 mL) and brine (40 mL), dried over anhydrous Na_2_SO_4_ and filtered. The filtrate was then evaporated under reduced pressure to afford compound 3 as a white pure crystalline solid (2.0 g, quantitative yield). Compound 3 was used in the next step without further purification. Mp: 129–131 °C; ${\left[a\right]}_{D}^{25}$ = −39° (c = 0.0054 g/mL, CHCl_3_); R_F_: 0.46, (petroleum ether 40–60 °C/EtOAc 80:20); ^1^H NMR (600 MHz, CDCl_3_): *δ* 0.06 (s, 6H, Si(CH_3_)_2_), 0.78 (s, 3H), 0.89 (s, 9H, C(CH_3_)_3_), 1.02 (s, 3H), 1.19–2.43 (m, 18H), 3.43–3.53 (m, 1H, 3*α*-H), 4.09 (dd, *J* = 12.2, 6.3 Hz, 1H, 21-H), 4.15 (dd, *J* = 12.0, 7.4 Hz, 1H, 21-H) 5.25 (t, *J* = 6.7 Hz, 1H, 20-H), 5.32 (d, *J* = 5.1 Hz, 1H, 6-H); ^13^C NMR (75 MHz, CDCl_3_): *δ* −4.4, 14.3, 18.4, 18.7, 19.6, 21.1, 21.2, 24.5, 26.1, 26.3, 31.8, 31.9, 32.2, 35.8, 36.9, 37.5, 42.9, 44.0, 50.7, 72.7, 115.7, 121.0, 141.8, 155.9; ESI-HRMS: *m*/*z* [M +Na]^+^ calcd. for C_27_H_46_O_2_SiNa 453.31584, found 453.3158.

### 2.5. Synthesis of (17S,20S)-3β-(t-Butyldimethylsilyloxy)-17α,20-Methan-5-Pregnane-21-ol (4)

To a solution of compound 3 (1.8 g, 4.17 mmol) in anhydrous toluene (33.6 mL) was added at 25 °C diiodomethane (CH_2_I_2_) (1.8 mL, 21.6 mmol). The reaction mixture was then cooled to −78 °C and a solution of diethylzinc (0.9 M in hexane, 24 mL, 21.6 mmol) was added. The reaction mixture was then stirred at 25 °C for 2 h. After completion of the reaction (monitored by ^1^H NMR), the reaction mixture was quenched using 10% aqueous HCl at 0 °C until pH 5.5, followed by extraction using EtOAc (30 mL × 3 times). The organic layer was washed with brine, dried over anhydrous Na_2_SO_4_ and the solvent was removed in vacuo. The residue was purified by FCC (petroleum ether 40–60 °C/EtOAc 90/10 → 85/15) to obtain compound 4 (1.1 g, 60% yield). Mp: 154–157° C; ${\left[a\right]}_{D}^{25}$ = −50° (c = 0.0036 g/mL, CHCl_3_); R_F_: 0.46 (petroleum ether 40–60 °C/EtOAc 80:20); ^1^H NMR (600 MHz, CDCl_3_): *δ* 0.06 (s, 6H, Si(CH_3_)_2_), 0.78 (s, 3H), 0.89 (s, 9H, C(CH_3_)_3_), 0.90–0.97 (m, 1H) 1.01 (s, 3H), 1.03–2.32 (m, 20H), 3.45–3.54 (m, 2H), 3.58–3.66 (m, 1H, 22-H), 5.33 (d, *J* = 5.3 Hz, 1H, 6-H); ^13^C NMR (75 MHz, CDCl_3_): *δ −*4.4, 16.3, 17.1, 18.4, 19.6, 19.8, 20.6, 25.2, 26.1, 29.1, 32.1, 32.2, 32.7, 33.3, 36.3, 36.8, 37.5, 41.1, 42.9, 50.6, 54.9, 65.3, 72.7, 121.1, 141.7; ESI-HRMS: *m*/*z* [M + Na]+ calcd. for C_28_H_48_O_2_SiNa 467.3314, found 467.3314.

### 2.6. Synthesis of (17S,20S)-3β-(t-Butyldimethylsilyloxy)-17α,20-Methan-5-Pregnane-21-al (5)

To a solution of compound 4 (0.85 g, 1.91 mmol) in dry CH_2_Cl_2_ (100 mL) was added at 0 °C Dess–Martin periodinane (1.62 g, 3.82 mmol) and the reaction mixture was stirred at 25 °C for 1.5 h. After completion of the reaction (checked by TLC), a mixture of saturated aqueous NaHCO_3_ and 10% aq. Na_2_S_2_O_4_ (1:1) was added and the resulting mixture was stirred for 30 min. The reaction mixture was extracted with diethyl ether (Et_2_O) (30 mL × 3 times) and the combined organic layers washed with saturated aqueous NaHCO_3_ and brine and dried over Na_2_SO_4_. The solvent was then removed in vacuo. The residue was purified by FCC (petroleum ether 40–60 °C/EtOAc 98/2 → 96/4) to afford compound 5 as a white crystalline solid (0.72 g, yield = 84%). Mp: 114–116 °C; ${\left[a\right]}_{D}^{25}$ = −21° (c = 0.0056 g/mL, CHCl_3_); R_F_: 0.78 (petroleum ether 40–60 °C/EtOAc 80:20); ^1^H NMR (600 MHz, CDCl_3_): *δ* 0.05 (s, 6H, Si(CH_3_)_2_), 0.80 (s, 3H), 0.88 (s, 9H, C(CH_3_)_3_), 0.92–0.99 (m, 1H), 1.00 (s, 3H), 1.04–2.30 (m, 21H), 3.42–3.52 (m, 1H, 3*α*-H), 5.33 (d, *J* = 5.1 Hz, 1H, 6-H), 9.08 (d, *J* = 6.2 Hz, 1H, CHO); ^13^C NMR (75 MHz, CDCl_3_): *δ -*4.4, 17.1, 18.4, 19.6, 20.5, 21.1, 25.3, 26.0, 29.6, 31.0, 32.0, 32.1, 32.6, 33.1, 36.8, 37.5, 42.3, 42.9, 43.8, 50.4, 53.9, 72.6, 120.9, 141.7, 202.0; ESI-HRMS: *m*/*z* [M + Na]^+^ calcd. for C_28_H_46_O_2_SiNa 465.31638, found 465.3164.

### 2.7. Synthesis of (((2′R,3S,17S)-2′-(2-Bromovinyl)-5-Androstene-17,1′-Cyclopropan)-3-yl)oxy)(Tert-Butyl)Dimethylsilane (6)

To a round bottom flask (bromomethyl)triphenyl phosphonium bromide (473 mg, 1.09 mmol) was added and the mixture was cooled to 0 °C. A solution of NaHMDS (1M in THF, 1.1 mL, 1.09 mmol) was added at 0 °C under argon and the reaction was stirred for 20 min. A solution of aldehyde 5 (120 mg, 0.27 mmol) in dry THF (0.5 M) was added dropwise at 0 °C and the reaction mixture was stirred for 1 h at the same temperature. The reaction was quenched with water at 0 °C and the reaction mixture was extracted using EtOAc (20 mL 3× times). The organic layer was washed with brine, dried over Na_2_SO_4_ and the solvent was removed in vacuo. The residue was purified by FCC (petroleum ether 40–65 °C/EtOAc 99/1) to afford compound 7 as a white crystalline solid (*E,Z* mixture 1:1, 116 mg, 83% yield). Mp: 126–129 °C; ${\left[a\right]}_{D}^{25}$ = −50° (c = 0.0024 g/mL, CHCl_3_); R_F_: 0.88 (petroleum ether 40–65 °C/EtOAc 95/5); ^1^H NMR (600 MHz, CDCl_3_): *δ* 0.06 (s, 6H, Si(CH_3_)_2_), 0.25 (t, *J* = 5.1 Hz) and 0.30 (t, *J* = 4.9 Hz) (1H), 0.75 (s, 3H), 0.81 (s, 3H), 0.89 (m, 9H), 1.00 (s) and 1.01 (s) (3H), 1.12–2.28 (m, 21H), 3.49–3.53 (m, 1H, 3*α*-H) 5.32 ( *J* = 3.0 Hz, 1H), 5.64 (dd, *J* = 7.09, 9.3 Hz) and 5.80 (dd, *J* = 9.6, 13.4 Hz) (1H), 5.97 (d, *J* = 13.4 Hz, 1H) and 6.08 (d, *J* = 7.00 Hz) (1H); ^13^C NMR (300 MHz, CDCl_3_): *δ* −4.4, 16.7, 17.1, 18.4, 19.6, 19.7, 19.9, 20.6, 20.9, 21.6, 25.2, 25.3, 26.1, 29.9, 30, 32.1, 32.2, 32.6, 32.7, 33.4, 36.9, 37.6, 39.4, 41.6, 41.7, 42.9, 50.5, 50.6, 54.8, 54.9, 72.7, 101.1, 105.3, 121.2, 137.0, 140.2, 141.8; APCI-HRMS: *m*/*z* [(M-OTBS) + H]^+^ calcd. for C_23_H_33_^79^BrOSi 389.1662, found 389.1662.

### 2.8. Synthesis of (2′R,3S,17S)-2′-(2-Bromovinyl)-5-Androstene-17,1′-Cyclopropan-3-ol (ENT-A013)

To a solution of compound 6 (112 mg, 0.21 mmol) in anhydrous CH_2_Cl_2_ (6 mL) HF·pyridine (0.3 mL) was added at 0 °C and the reaction mixture was stirred at 25 °C for 2 h. The reaction was quenched with water at 0 °C, and the resulting mixture was extracted using CH_2_Cl_2_ (10 mL × 2 times). The organic layer was washed with brine, dried over Na_2_SO_4_ and the solvent was removed in vacuo. The residue was purified by FCC (elution solvent: Hexane/EtOAc 90/10 → 85/15) to afford compound ENT-A013 as a solid (*E,Z* mixture 1:1, 85 mg, in quantitative yield). Mp: 83–86 °C; ${\left[a\right]}_{D}^{25}$ = −44° (c = 0.0034 g/mL, CHCl_3_); R_F_: 0.22 (Hexane/EtOAc 80:20); ^1^H NMR (600 MHz, CDCl_3_): *δ* 0.25 and 0.30 (two t, *J* = 5.1 and 4.9 Hz, 1H), 0.75 and 0.81 (two s, 3H), 1.01 and 1.02 (two s, 3H), 1.04–2.33 (m, 21H), 3.48–3.59 (m, 1H, 3*α*-H) 5.36 (d, *J* = 5.7 Hz, 1H), 5.65 and 5.80 (two dd, *J* = 9.4, 7.0 Hz and *J* = 13.4, 9.5 Hz, 1H), 5.97 (d, *J* = 13.5 Hz) and 6.09 (d, *J* = 7.0 Hz) (1H); ^13^C NMR (300 MHz, CDCl_3_): *δ* 16.7, 17.1, 19.6, 19.7, 19.9, 20.6, 20.8, 21.5, 25.1, 25.2, 29.9, 30, 31.7, 32, 32.1, 32.5, 32.6, 33.3, 36.7, 37.4, 38.5, 39.4, 41.6, 41.7, 42.4, 50.3, 50.4, 54.7, 54.8, 71.8, 101.1, 105.3, 121.7, 137, 140.2, 140.9; APCI-HRMS: *m*/*z* [(M-OH) + H]^+^ calcd. for C_23_H_33_^79^Br 387.1682, found 387.1687; *m*/*z* [(M-OH) + H]^+^ calcd. for C_23_H_33_^81^Br 389.1661, found 389.1664.

The final compound ENT-A013, obtained as *E,Z* mixture (1:1), was then subjected to chiral high-performance liquid chromatography (HPLC) separation to obtain the pure geometrical isomers ENT-A013*E* and ENT-A013*Z*. Chiral HPLC conditions: Kromasil Chiral 10-TBB mm × 250 mm column, eluting with hexane/iso-propanol (i-PrOH) 90:10 isocratic mixture, employing refractive index detection, flow rate: 1.5 mL/min. ENT-A013*E* retention time *t_R_* = 16.66 min, ENT-A013*Z* retention time *t_R_* = 17.94 min.

ENT-A013*E*: Mp: 76–80 °C; R_F_: 0.23 (Hexane/EtOAc 80:20); ${\left[a\right]}_{D}^{25}$= +7° (c= 0.0012 g/mL, CHCl_3_); ^1^H NMR (600 MHz, CDCl_3_): *δ* 0.25 (t, *J*= 4.9 Hz, 1H), 0.75 (s, 3H), 1.01 (s, 3H), 1.12–2.28 (m, 21H), 3.44–3.59 (m, 1H, 3*α*-H), 5.36 (d, *J*= 4.2 Hz, 1H), 5.80 (dd, *J* = 13.4, 9.3 Hz, 1H), 5.97 (d, *J* = 13.4 Hz, 1H); ^13^C NMR (300 MHz, CDCl_3_): *δ* 16.5, 19.4, 19.7, 20.4, 21.4, 25.04, 29.7, 31.6, 31.9, 32.4, 33.2, 36.6, 37.2, 38.4, 41.5, 42.7, 50.2, 54.7, 71.7, 100.9, 121.6, 140.09, 140.7.

ENT-A013*Z*: Mp: 86–89 °C; R_F_: 0.2 (Hexane/EtOAc 80:20); ${\left[a\right]}_{D}^{25}$= −108° (c = 0.0013 g/mL, CHCl_3_); ^1^H NMR (600 MHz, CDCl_3_): *δ* 0.30 (t, *J* = 5.06 Hz, 1H), 0.81 (s, 3H), 1.00 (s, 3H), 1.12–2.28 (m, 21H), 3.49–3.53 (m, 1H, 3*α*-H) 5.32 (d, *J* = 3.0 Hz, 1H), 5.64 (dd, *J* = 7.1, 9.3 Hz, 1H), 5.80 (dd, *J*= 9.6, 13.4 Hz, 1H), 6.09 (d, *J* = 7.0 Hz, 1H); ^13^C NMR (300 MHz, CDCl_3_): *δ* 16.9, 19.4, 19.5, 20.6, 21.4, 25.1, 29.8, 31.6, 31.9, 32.3, 33.2, 36.6, 37.2, 39.2, 41.5, 42.3, 50.3, 54.6, 71.7, 105.1, 121.6, 140.04, 140.8.

### 2.9. Cell Lines

PC12 and HEK293T cells were obtained from LGC Promochem GmbH (Teddington, UK) and cultured under the conditions specified by the manufacturer. NIH3T3 cells were stably transfected with human TrkB plasmid. PC12 cells were grown in DMEM medium (11965084, Gibco, Grand Island, NY, USA) containing 10% Horse Serum (16050122, Gibco, Grand Island, NY, USA), 5% Fetal Bovine Serum (10270106, Gibco, Grand Island, NY, USA), 100 units/mL Penicillin and 0.1 mg/mL Streptomycin (15140122, Gibco, Grand Island, NY, USA) at 5% CO_2_ and 37 °C. Cells were used between passages 5–20. HEK293T and NIH3T3 cells were grown in the same medium as PC12 cells supplemented with 10% Fetal Bovine Serum and lacking Horse Serum. For transient transfection of HEK293T cells, lipofectamine was used and cells were transfected with human p75^NTR^ and Traf6 plasmids. To determine the intracellular activity of endogenous secretases, we tagged (tGFP) a truncated mutant (Tm) of APP containing the original amino acids sequence from M516 to N773. This Tm conserves the secretases cleavage sites, clathrin binding site, tau protein binding site, collagen binding site and some glycosylation sites critical for protein localization and intracellular trafficking. The cDNA of the TmAPP gene was amplified by PCR and subcloned into pturbo-GFP vector. The construction was transfected into U2OS cells (ATCC Cat Number HTB-96) using Lipofectamine LTX (Thermo Fisher Scientific, Waltham, MA, USA). After 48 h, individual cells were plated in MTP96 plates using F.SIGHT (CYTENA GmbH, Freiburg, Germany), and cultured in complete medium containing 250 μg/mL G418 for selection. The selection medium was changed every two to three days. The cells were allowed to grow for 3 to 4 weeks, after which clones were isolated for further analysis. Resistant clones were checked by RT-PCR and epifluorescence microscopy.

### 2.10. Immunoprecipitation and Immunoblotting

For immunoprecipitation experiments PC12 or HEK293T, cells were used when at 70–80% confluent. Cells were starved of serum for 4 h and subsequently treated with 100 ng/mL NGF or 500 nM of compound ENT-A013 for 30′. Cells were then lysed in Pierce™ IP Lysis Buffer (87788, Thermo Fischer Scientific, Waltham, MA, USA) containing proteases (539138, Calbiochem, Darmstadt, Germany) and phosphatase inhibitors (524629, Calbiochem, Darmstadt, Germany). Lysates were then immunoprecipitated overnight at 4 °C with TrkA antibody (1:100, 06-574, Sigma-Aldrich, St. Louis, MO, USA) (Table S1) or p75^NTR^ antibody (1:100, ab6172, Abcam plc., Cambridge, UK) followed by 4 h incubation with protein G-plus agarose beads (sc-2002, Santa Cruz Biotechnology, Inc., Dallas, TX, USA). Beads were then collected, washed 3X with lysis buffer, resuspended in SDS loading buffer and subjected to Western blot against phosphorylated Tyrosine (1:1000, BAM1676, R&D Systems, Inc., Minneapolis, MN, USA) or Traf6 antibody (1:2000, ab33915, Abcam, plc., Cambridge, UK). Whole cell lysates were subjected to Western blot against TrkA (1:1000, 06-574, Sigma-Aldrich, St. Louis, MO, USA), phosphorylated TrkB (1:1000, ABN1381, Sigma-Aldrich, St. Louis, MO, USA), phosphorylated TrkC (1:1000, STJ90960, St John’s Laboratory, Ltd., London, UK), TrkB (1:1000, 07-225-I, Sigma-Aldrich, St. Louis, MO, USA), TrkC (1:1000, C44H5, Cell Signaling Technology, Danvers, MA, USA), p75 (1:1000, 839701, Biolegend, Inc., San Diego, CA, USA), Traf6 (1:2000, ab33915, Abcam, plc., Cambridge, UK) phosphorylated Akt (1:1000, 9721S, Cell Signaling Technology, Danvers, MA, USA), phosphorylated Erk1/2 (1:1000, 9101S, Cell Signaling Technology, Danvers, MA, USA), total Akt (1:1000, 4691S Cell Signaling Technology, Danvers, MA, USA) and total Erk1/2 (1:1000, 4695S, Cell Signaling Technology, Danvers, MA, USA).

### 2.11. CellTox Assay

CellTox assay (G8742, Promega Corporation, Maddison, WI, USA) was used to assess survival of PC12 cells under serum deprivation conditions. PC12 cells were plated in 96-well plates, starved of serum for 4 h and subsequently treated with NGF (100 ng/mL) or ENT-A013 (500 nM) in the presence or absence of TrkA inhibitor GW441756 (20 μM, G-190, Alomone labs, Jerusalem, Israel) for 24 h. CellTox assay reagents and Hoescht (1:10,000, H3570, Invitrogen, Waltham, MA, USA) were added to each well for 30′ and cells were then imaged with a Zeiss AXIO Vert A1 fluorescent microscope (Zeiss, Jena, Germany). CellTox positive cells were normalized to total number of cells for each image.

### 2.12. Primary Dorsal Root Ganglia Neurons

Primary Dorsal Root Ganglia (DRG) neurons were isolated from P0-P1 mouse pups (C57BL/6J, The Jackson Laboratory, Bar Harbor, ME, USA) as described previously [18] and cultured with Neurobasal medium (21103049 Gibco, Grand Island, NY, USA) containing 2% B27 (17504044, Gibco, Grand Island, NY, USA), 100 ng/mL NGF (N-100, Alomone Labs, Jerusalem, Israel), 1X GlutaMax (35050061, Gibco, Grand Island, NY, USA), 10 mM HEPES (15630080, Gibco, Grand Island, NY, USA), 1% Penicillin/Streptomycin (15070063, Gibco, Grand Island, NY, USA) and 10 μM 5-Fluoro-2′-deoxyuridine (343333, Calbiochem, Darmstadt, Germany). For TUNEL assay, cells were cultured for 14 days in vitro and then the medium was deprived of NGF before adding ENT-A013 (500 nM, every 24 h) along with NGF-neutralizing antibody (N8773, 1:500, Sigma-Aldrich, St. Louis, MO, USA). Cells were then incubated for 48 h before being fixed with 4% PFA and labelled with TUNEL (11684795910, F. Hoffmann–La Roche Ltd., Basel, Switzerland) following the manufacturer’s protocol. Subsequently, cells were immunostained against Tuj1 (1:2000, 801201, Biolegend, Inc., San Diego, CA, USA) and anti-mouse Cy3 (1:1000, Invitrogen, A10521, Waltham, MA, USA) and imaged with a Leica SP8 confocal microscope. For neurite outgrowth assay, cells were cultured for 5 days in vitro with medium deprived of NGF in the presence of ENT-A013 (500 nM, every 48 h), along with NGF-neutralizing antibody. Subsequently, cells were fixed with 4% paraformaldehyde, immunostained against TUJ1 and anti-mouse Alexa fluor 488 (1:1000, A-11029, Thermo Fischer Scientific, Waltham, MA, USA). Cells were then imaged by confocal microscopy and neurite length was measured using Fiji software (NIH, Bethesda, Maryland, USA).

### 2.13. Primary Hippocampal Neurons

Primary hippocampal neurons were isolated from E17.5 mouse embryos (C57BL/6J, The Jackson Laboratory, Bar Harbor, ME, USA) as previously described [19] and cultured with Neurobasal medium containing 2% B27, 1X GlutaMax, 10 mM HEPES and 1% PenStrep. Cells were maintained in a humified incubator at 37 °C and 5% CO_2_. For TUNEL assay, cells were left in culture for 16–18 days and then treated with 5 μM oligomeric Aβ along with ENT-A013 (500 nM, every 24 h) for 48 h. Cells were then fixed with 4% PFA and labelled with TUNEL (F. Hoffmann–La Roche Ltd., Basel, Switzerland, cat# 11684795910) following the manufacturer’s protocol. Subsequently, cells were immunostained against Tuj1 and imaged with confocal microscopy. For synaptic plasticity assay, cells were left in culture for 16–18 days and then treated with 5 μM oligomeric Aβ along with ENT-A013 (500 nM) for 4 h. Cells were then fixed with 4% PFA and immunostained against Tuj1 and Synaptophysin (1:1000, PA1-1043, Invitrogen, Waltham, MA, USA), anti-mouse Alexa fluor 488 (1:1000, A-11029, Invitrogen, Waltham, MA, USA) and anti-rabbit Alexa fluor 546 (1:1000, A10040, Invitrogen, Waltham, MA, USA). Images were acquired using confocal microscopy and the total area of synaptophysin positive puncta was measured and normalized to Tuj1 total area.

### 2.14. Preparation of Aβ Oligomers

Aβ (1–42) peptide was purchased from AnaSpec (AS-20276, AnaSpec, Fremont, CA, USA) and prepared according to manufacturer’s instructions. For oligomeric Aβ treatment, Aβ peptide was diluted in DMEM at the specified concentrations and left for 24 h at 37 °C. It was then centrifuged for 5′ at 15,000× *g* and the supernatant collected as oligomeric Aβ as previously described [20].

### 2.15. Electrophysiology

C57BL/6J mice were euthanized under halothane anesthesia and the brain was quickly dissected and placed in ice-cold oxygenated artificial cerebrospinal fluid (aCSF; 95% O_2_/5% CO_2_) containing (in mM): 125 NaCl, 3.5 KCl, 26 NaHCO_3_, 1 MgCl_2_ and 10 glucose (pH = 7.4, 315 mOsm/L). The brain was then placed onto the stage of a vibratome (Leica, VT1000S, Leica Biosystems GmbH, Wetzlar, Germany) and transverse slices containing the hippocampus were obtained. The brain slices (400 μM) were then placed in a submerged chamber containing oxygenated (95% O_2_/5% CO_2_) aCSF (in mM: 125 NaCl, 3.5 KCl, 26 NaHCO_3_, 2 CaCl_2_, 1 MgCl_2_ and 10 glucose (pH = 7.4, 315 mOsm/L)) at room temperature and left resting for at least 1 hr. Following that, slices were treated for 3–5 h with oligomeric Aβ_1–42_ (5 μM) in the presence or absence of compound ENT-A013 (500 nM) and subsequently placed in the recording chamber. A recording electrode, containing 3M NaCl was placed in the stratum radiatum of the CA1 area of the hippocampus and a platinum/iridium metal stimulating microelectrode (Harvard Apparatus, Holliston, MA, USA) was placed in the same layer, 300 μM apart. Field excitatory post-synaptic potentials (fEPSP) were amplified using an EXT-02F amplifier (National Instruments Corporation, Austin, TX, USA), digitized with ITC-18 (HEKA Elektronik GmbH, Reutlingern, Germany) and recorded in a computer running Windows 10 with IgorPro software (Wavemetrics Inc., Portland, OR, USA). Electrical stimulation was generated using a stimulator equipped with a stimulus isolation unit (NPI Electronic GmbH, Tamm, Germany). To induce LTP, theta-burst stimulation (TBS), consisting of 5 pulses at 100 Hz, was given at theta-rhythm (every 200 ms) and repeated three times with an inter-stimulus interval of 20 s. The fEPSP amplitude was calculated from the minimum voltage value of the synaptic response (3–5 ms following stimulation) compared to the baseline voltage prior to stimulation. fEPSP recordings were acquired every 90″ for 10′ before TBS and continued for approximately 50′ following LTP induction. Recorded traces were analyzed using a custom-made script in the IgorPro software (Wavemetrics Inc., Portland, OR, USA).

### 2.16. Metabolic Stability

Human liver microsome (pooled) concentration was set at 0.5 mg/mL and 1 mM NADPH served as a cofactor for oxidative (CYP-mediated) metabolic stability profiling. ENT-A013 was tested at 1 μM. Incubation conditions ensured linear metabolite formation with respect to reaction time and protein concentration. Incubations took place in triplicates at 37 °C in the presence of negative and positive controls (low vs. rapid clearance). Each reaction was terminated after the specified incubation period (60 min) and plates were read on Lionheart FX (BioTek, Winooski, VT, USA). Residual (%) of time zero (ENT-A013 depletion) was determined.

### 2.17. Isozyme-Specific CYP450-Metabolism

Baculosomes^®^ expressing CYP1A2, CYP2A6, CYP2B6, CYP2C9, CYP2C19, CYP2D6 and CYP3A4 human CYP450 isoenzymes were purchased from Thermo Fisher Scientific (Waltham, MA, USA). All reagents were handled and prepared according to the manufacturer’s protocol. ENT-A013 was tested at 1 μM. Enzymatic activity in the presence of ΕΝΤ-A013 was assessed based on the kinetic model for CYP1A2, CYP2A6, CYP2B6, CYP2C9, CYP2C19, CYP2D6 and CYP3A4 isoforms. Reactions took place in triplicates at 20 °C. Glucose-6-phosphate at 333 mM and glucose-6-phosphate-dehydrogenase at 30 U/mL in 100 mM potassium phosphate, pH 8.0 (regeneration system) converted NADP^+^ (10 mM in 100 mM potassium phosphate, pH 8.0) into NADPH to initiate the CYP450 reaction. Upon the addition of the fluorescent substrate, immediately (<2 min), the fluorescence signal was monitored over time at suitable excitation and emission wavelengths by Lionheart FX (BioTek, Winooski, VT, USA) (Table S2). CYP450 inhibition (%) was determined based on the reaction rates (fluorescence intensity changes per unit time). A total of *n* = 60 measurements per minute were acquired for a total time of t = 60 min.

% Inhibition= (1 − X/A) × 100%(1)

where: X is the rate observed in the presence of test-compound; A is the rate observed in the presence of negative (solvent, DMSO) control.

### 2.18. Molecular Modelling and Docking Studies

The crystal structure of the complex of TrkA immunoglobulin-like domain 5 (D5) homodimer with the mature NGF homodimer (PDB ID: 1WWW) [21] was used for modeling studies. The structure of NGF has the following missing residues: Ser 122, Pro 182—Ser 187 and Ala 237—Ala 241 (residue numbers correspond to the UniProt sequence of human NGF, UniProt code: P01138). The missing residues were built with the AutoModel class of MODELLER v.9.23 [22]. The linker corresponding to residues Pro 182—Ser 187 has a conserved conformation in existing crystal structures and therefore it was built using the structure of human NGF from PDB ID: 5JZ7 [23] as a template. The final model was prepared with the Protein Preparation Wizard [24,25] of Schrödinger Suite v.2020. Specifically, bond orders were assigned, hydrogens were added, disulfide bonds were created, protonation states were assigned at pH 7 with PROPKA [26] and optimization of the H-bond network and restrained energy minimization using the OPLS3e force field [27] with heavy atoms restricted to a maximum root mean squared deviation (RMSD) from the initial structure of 0.3 Å were performed. The SiteMap tool [28,29,30] of the Schrödinger Suite was used for identification of possible binding sites in the structure. From the predicted binding sites, sites 1a and 1b, previously described [17] as the possible binding sites of the TrkA agonist BNN27, were used for docking studies. The two isomers, ENT-A013E and ENT-A013Z, were built in Maestro [31] and prepared with the LigPrep tool [32] of the Schrödinger Suite. The compounds were docked to the two symmetric sites 1b of the TrkA-NGF complex using the standard precision (SP) protocol of the Glide tool [33,34,35,36] of the Schrödinger Suite. Grids were generated with Glide to describe the physicochemical properties of the protein pockets. The hydroxyl groups of serine, threonine and tyrosine residues and thiol groups of cysteine residues, located within the grid box that describes the binding sites, were allowed to rotate during the docking calculation, to capture all possibilities for making hydrogen bonds with the ligands. For site 1b, the Glide SP scores for the two compounds were about −3 kcal/mol, indicating modest binding affinity. For site 1a, which is narrower than 1b, ligand poses with favorable scores could not be obtained with rigid body docking and, therefore, the Induced Fit Docking (IFD) tool [37,38,39,40] of the Schrödinger Suite was used; this utilizes Glide for docking and Prime [41,42,43] for protein remodeling, and resulted in docking poses with docking scores of about −10 kcal/mol. Graphical analysis of the docking poses was carried out with VMD v.1.9.3 [44].

### 2.19. Statistical Analysis

All values are expressed as the mean ± s.e.m. Mann–Whitney U or Student’s *t*-test was used for the comparison of two groups. One-way ANOVA followed by Tukey’s multiple-comparison test was used for multiple group comparisons. Where fold change values are used, statistical analysis was performed in log2 transformed values to ensure a normally distributed dataset. A *p* < 0.05 was considered to mark statistical significance. Statistical analysis was performed using GraphPad Prism 7 (GraphPad Software Inc., San Diego, CA, USA).

## 3. Results

### 3.1. Chemistry

**Scheme 1:**
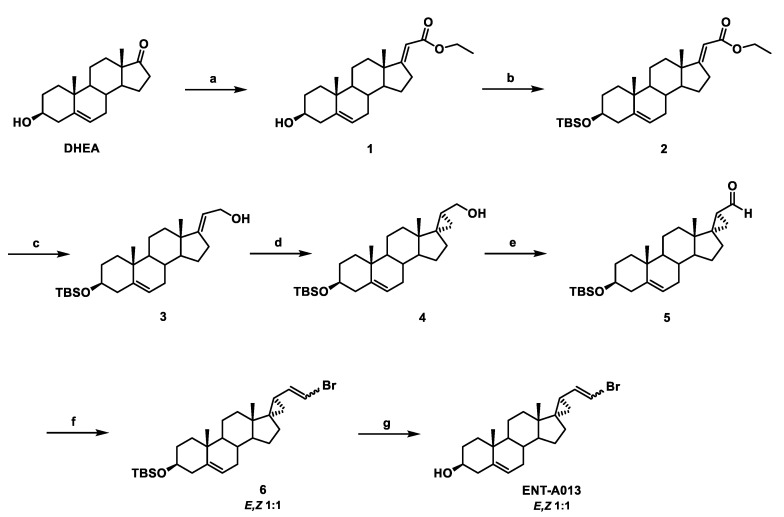
Synthesis of ENT-A013 (mixture of *E,Z* isomers 1:1). Reagents and conditions: (**a**) (CH_3_CH_2_O)_2_P(O)CH_2_C(O)OCH_2_CH_3_, EtONa, THF/EtOH 1:1, reflux, overnight; (**b**) TBDMSCl, Imidazole, I_2,_ THF, 0 °C to 25 °C, overnight; (**c**) DIBAL-H, CH_2_Cl_2_, −78 °C, 2.5 h; (**d**) CH_2_I_2_, Et_2_Zn, −78 °C to 25 °C, 1 h; (**e**) Dess–Martin periodinane, CH_2_Cl_2_, 0 °C to 25 °C, 1.5 h; (**f**) [Ph_3_PCH_2_Br]Br, NaHMDS (1 M in THF), THF, 0 °C, 1 h; (**g**) HF^.^Pyridine, CH_2_Cl_2_, 0 °C to 25 °C, 0.5 h.

**Scheme 2:**
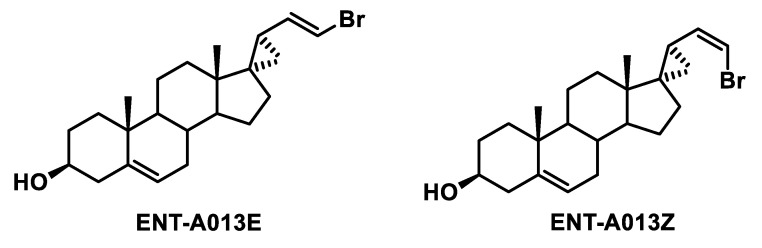
Structures of ENT-A013 geometrical isomers (ENT-A013E and ENT-A013Z) obtained after HPLC separation.

The synthesis of ENT-A013 is reported in Scheme 1 and involves eight high-yielding steps from dehydroepiandrosterone (DHEA). Thus, a Horner–Emmons reaction of DHEA with triethyl phosphonoacetate in the presence of sodium ethoxide (EtONa) afforded the (*E*)-*a,b*-unsaturated ester 1 in 96% yield, which was then reacted with *tert*-butyldimethylsilyl chloride (TBDMS-Cl) to afford the TBDMS-protected alcohol 2 in 93% yield. Selective reduction of the ester group in 2 using diisobutylaluminum hydride (DIBAL-H) gave the allylic alcohol 3 in quantitative yield, which was subjected to a Simmons–Smith cyclopropanation reaction in the presence of diiodomethane and diethylzinc to yield the (17*S*,20*S*)*-*cyclopropyl derivative 4 in 60% yield [45]. Compound 4 was subsequently oxidized with Dess–Martin periodinane in dichloromethane (DCM) to afford the corresponding aldehyde 5 in 84% yield. A Wittig reaction of aldehyde 5 with (bromomethyl)triphenylphosphonium bromide in the presence of sodium bis(trimethylsilyl)amide solution (1 M in THF) (NaHMDS) afforded the vinyl-bromide derivative 6 (1:1 mixture of *E*,*Z* isomers) in 83% yield [46]. Deprotection of the C3 alcohol in 6 using HF^.^Pyridine complex in dry CH_2_Cl_2_ afforded ENT-A013 (mixture of *E,Z* isomers 1:1) in quantitative yield (Scheme 1). The mixture of ENT-A013 *E,Z* isomers (1:1) was subjected to chiral HPLC separation and the pure *E* and *Z* geometrical isomers, compounds ENT-A013E and ENT-A013Z, respectively, were isolated (Scheme 2). For the purposes of clarity in the studies below, when ENT-A013 is mentioned ENT-A013 [*E,Z* (1:1) mixture] was used. In the case of the pure *E*, *Z* geometrical isomer codes ENT-A013E and ENT-A013Z are employed, respectively.

### 3.2. Two Possible Sites of Interaction for ENT-A013 Isomers (ENT-A013E and ENT-A013Z) with the TrkA/NGF Receptor Complex

**Figure 1:**
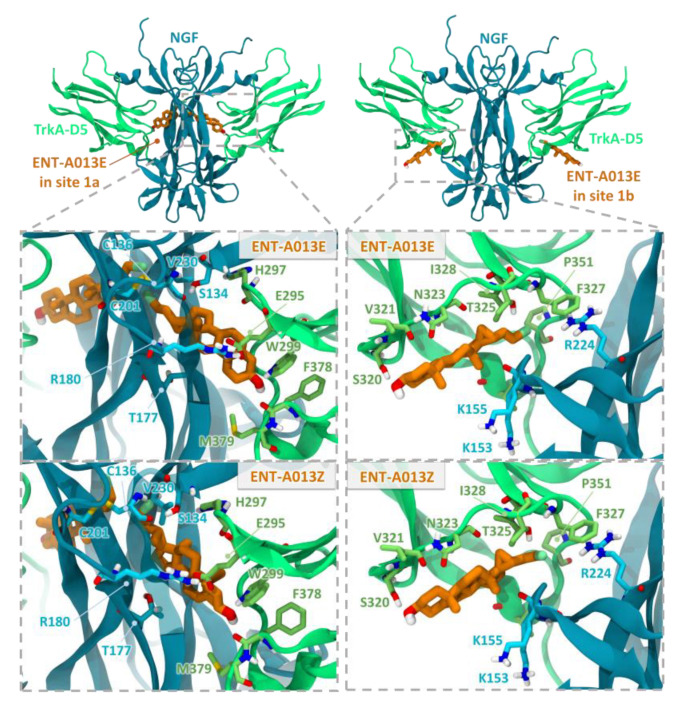
Docking poses of the two ENT-A013 isomers, ENT-A013E and ENT-A013Z, in sites 1a and 1b at the two symmetry-related TrkA-D5—NGF interfaces of the neurotrophin-receptor complex. The close-up views show residues lining the two sites. TrkA-D5 and NGF are shown in green and blue cartoon representation, respectively, with selected residues in stick representation colored by atom type. The compounds are shown in orange stick representation.

Computational docking studies were performed to investigate the mechanism of action of the two isomers, ENT-A013E and ENT-A013Z. The extracellular domain of TrkA has been previously reported to be a drug target for amitriptyline and MT2 TrkA agonists [47,48,49]. Prior Saturation Transfer Difference Nuclear Magnetic Resonance (STD-NMR) experiments have indicated that an analogue of ENT-A013, BNN27, binds at the interface of TrkA-D5 with NGF, thus bridging the heterodimer [17]. Specifically, two binding sites, site 1a and site 1b, found at the interfaces of the two proteins, were proposed to be the most probable [17]. The same putative binding sites were also identified in the present work and the two compounds were docked into these sites (Figure 1). The binding poses of both compounds in the two sites present complementarity to the binding pockets. For site 1b, which is partially solvent-exposed, the docking poses have the 3β-hydroxyl group pointing towards the solvent region and the C17 steroid substituents facing towards the interface of TrkA-D5 and NGF, thus providing a possible explanation for the selectivity of the substituents. The docking poses in site 1a present various possible arrangements of the steroidal core, indicating structurally less stable binding. This observation was corroborated by preliminary molecular dynamics simulations of the complexes, which showed a greater tendency to dissociate for compounds in site 1a than site 1b (data not shown). However, the diversity of binding poses in site 1a may offer an entropic advantage upon compound binding in this site. Overall, the docking studies suggest two possible interaction modes of compounds ENT-A013E and ENT-A013Z with TrkA, which could lead to enhanced receptor activation.

### 3.3. ENT-A013 Exhibits Slow Depletion in Human Liver Microsomes

Metabolic stability refers to the susceptibility of a test-compound to biotransformation and can be interpreted using several approaches, as test-compounds can be ranked in terms of their intrinsic clearance (CL_int_) and in vitro half-life (t_1/2_) values, or based on parent structure loss during metabolic reactions [50,51]. Herein, we chose the latter. Parent structure loss is classified as very fast (>80%), fast (50–80%), moderate (20–50%), slow (5–19%) or very slow (<5%). Such categories have been defined according to set criteria, namely, high metabolism (t_1/2_ value of <30 min), moderate metabolism (30 min < t_1/2_ value of <60 min) and low metabolism (t_1/2_ value of >60 min) [52,53,54]. ENT-A013 is slowly depleted showing a 93% residual of time zero at t = 60 min. Translating such finding to an intrinsic clearance prediction, ENT-A013 may correspond to low or medium intrinsic clearance classification bands. Based on a rearrangement of the well stirred model [55,56], classification bands can be used to categorize compounds into low, medium or high clearance, assuming an extraction ratio of 0.3 and 0.7 for the low and high boundaries, respectively. Next, this can be scaled to intrinsic clearance (µL/min/mg protein) using the relevant liver weights [57] and microsomal protein concentration [50,58,59] obtained from the literature and computational data. For humans, an CL_int_ < 8.6 μL/min/mg protein defines a low intrinsic clearance classification band, whereas an CL_int_ > 47.0 μL/min/mg protein defines a high intrinsic clearance classification band. Low clearance test-compounds are characterized by reduced doses, enhanced exposure and prolonged half-life, being suitable for once-daily dosing.

### 3.4. ENT-A013 Shows Weak-to-Moderate CYP Inhibition

**Figure 2:**
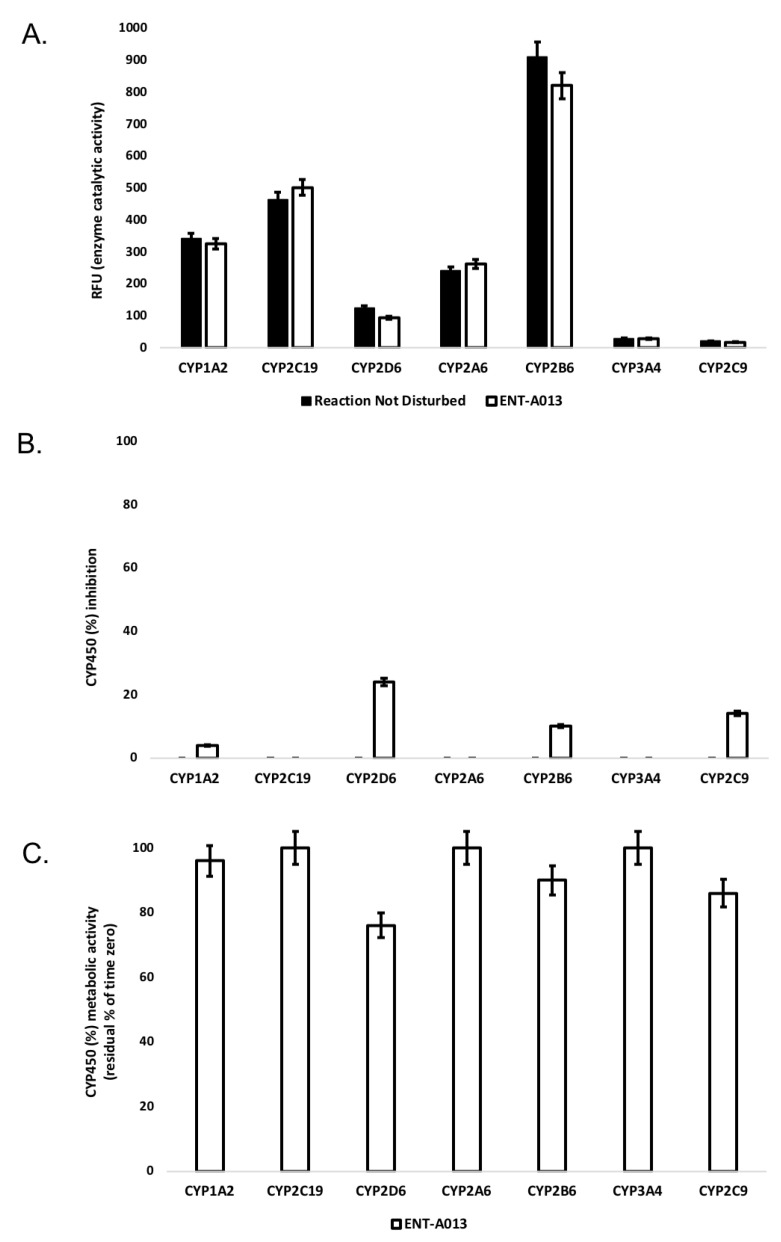
(**A**) Enzyme (catalytic) activity of the CYP1A2, CYP2A6, CYP2B6, CYP2C9, CYP2C19, CYP2D6 and CYP3A4 isoenzymes upon the administration of ΕΝΤ-A013 at 1 μΜ (60 min). RFU: relative fluorescence units. Reaction Not Disturbed: reaction without ΕΝΤ-A013. (**B**) CYP450 (%) inhibition of CYP1A2, CYP2A6, CYP2B6, CYP2C9, CYP2C19, CYP2D6 and CYP3A4 isoenzymes upon the administration of ENT-A013 at 1 μM (60 min). (**C**) CYP450 (%) metabolic activity of CYP1A2, CYP2A6, CYP2B6, CYP2C9, CYP2C19, CYP2D6 and CYP3A4 isoenzymes upon the administration of ENT-A013 at 1 μM (60 min).

Human liver cytochrome P450 (CYP450) enzymes play a fundamental role in xenobiotic biodegradation, metabolism and toxicity, as well as xenobiotic-xenobiotic and/or xenobiotic-host interactions [60]. When measuring the disappearance rate of ENT-A013 upon linear velocity conditions in vitro, findings can be further extrapolated to (a) in vivo hepatic clearance, (b) extraction ratio, and (c) the effect of hepatic first-pass metabolism to total oral bioavailability. Data allow for the identification of biodegradation, metabolic and toxicity liabilities early on and inform structure–activity relationships (SAR) [52,53,54]. For this, the activity of seven major human CYP450s (namely, CYP1A2, CYP2A6, CYP2B6, CYP2C9, CYP2C19, CYP2D6 and CYP3A4) was assessed upon the administration of ENT-A013 at 1 μM, allowing for a) oxidative (CYP-mediated) metabolic stability profiling and b) the identification of the enzyme metabolizing isoforms responsible (the test system consists of recombinant human CYP450 and CYP450 reductase; cytochrome b_5_ may also be present). As our model depicts direct interactions between the test-compound and CYP450 isoenzymes, no concentration-dependent effects were observed. CYP450 substrates are considered to alter enzyme activity by changing enzyme conformation, disrupting enzyme structure and/or functioning and/or blocking the enzyme active site [61,62]. First, the catalytic activity of the seven major human CYP450s (namely, CYP1A2, CYP2A6, CYP2B6, CYP2C9, CYP2C19, CYP2D6 and CYP3A4) was assessed upon ENT-A013 administration at 1 μM. Catalytic activity was determined based on the relative fluorescence of the enzymatic reaction product (Figure 2A). At this concentration, ENT-A013 did not decrease the enzyme (catalytic) activity of the seven major CYP450s tested herein. Next, CYP450 (%) inhibition was tested. No product inhibition or mechanism-based inactivation of the CYP450 isoenzymes in question was obtained. New chemical entities with poor solubility can show artificially low CYP450 inhibition and thus, we may overlook new chemical entities with potential drug–drug interaction toxicities. No solubility issues were reported herein. CYP450 enzyme inhibition may lead to unexpectedly high exposure of co-administered xenobiotics and hence, increase the risk for adverse effects. As depicted in Figure 2B, ENT-A013 is a weak inhibitor of CYP1A2, CYP2B6 and CYP2C9 isoforms, while it exhibits moderate inhibition for CYP2D6. CYP450 (%) metabolic activity was also determined, expressed as residual % of time zero, for each of the CYP450 isoforms tested. Figure 2C summarizes our findings, according to which ENT-A013 was overall metabolically stable (from t = 0 to t = 60 min).

### 3.5. ENT-A013 Activates TrkA and Its Downstream Signaling Kinases Akt and Erk1/2 but Not TrkB, TrkC or p75^NTR^ Neurotrophin Receptor

**Figure 3:**
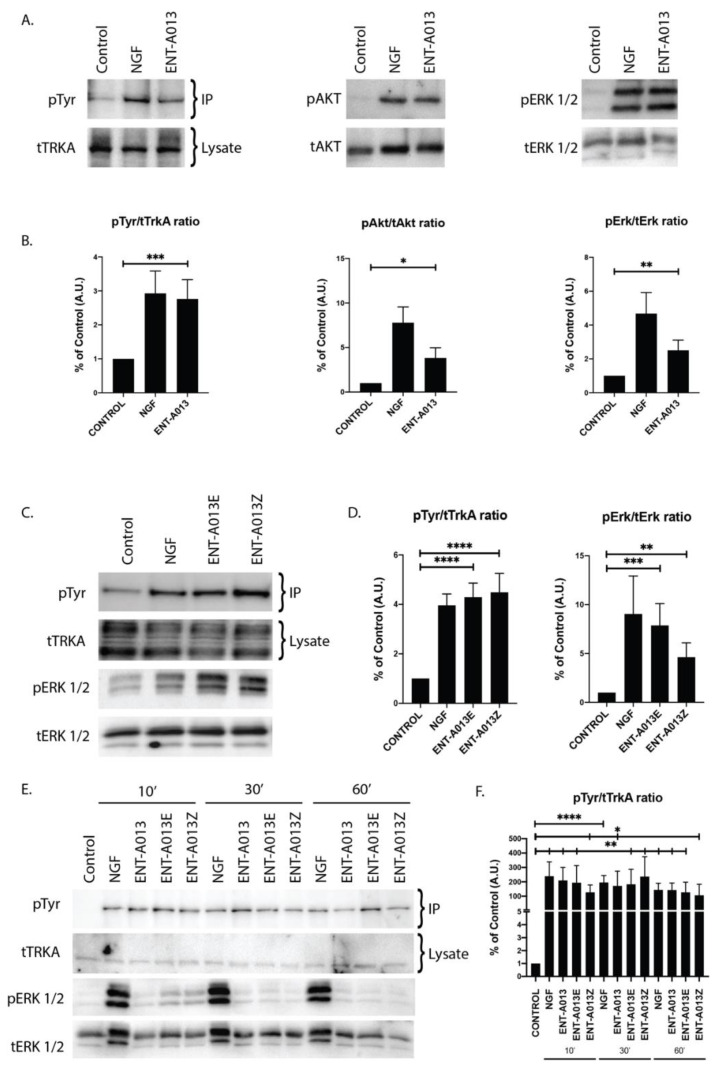
ENT-A013 ([E,Z (1:1) mixture] and the pure geometrical isomers ENT-A013E and ENT-A013Z activate TrkA and its downstream signaling kinases. (**A**,**B**) 30′ treatment of PC12 cells with ENT-A013 or vehicle was able to induce strong phosphorylation of TrkA and its downstream signaling kinases Akt and Erk1/2 as shown by immunoprecipitation or whole lysate Western blots. Representative blots of 3–6 independent experiments (**A**) and quantification (**B**) are shown. Data are shown as ±SEM; Student’s *t*-test against control:* p<0.05; ** *p* < 0.01; *** *p* < 0.001. (**C**,**D**) 30′ treatment of PC12 cells with ENT-A013E and Z or vehicle was able to induce phosphorylation of TrkA and Erk1/2 as shown by immunoprecipitation or whole lysate Western blot respectively. Representative blots of 4–6 independent experiments (**C**) and quantification (**D**) are shown. Data are shown as ±SEM; Student’s t-test against control: * ** *p* < 0.01; *** *p* < 0.001; **** p<0.0001. (**E**,**F**) PC12 cells treated for 10′, 30′ or 60′ with ENT-A013, ENT-A013E, ENT-A013Z or vehicle show that all compounds can induce TrkA phosphorylation, which is sustained over 60′ after treatment. Representative blots of 3 (IP) or 2 (Erk1/2) independent experiments (**E**) and quantification for pTyr activation (**F**) are shown. Data represent ±SEM; Student’s *t*-test against control: * *p* < 0.05; ** *p* < 0.01; **** *p* < 0.0001.

In order to test whether ENT-A013 activates the TrkA neurotrophin receptor and its downstream signaling kinases, we used PC12 cells, a cell line that express TrkA and p75^NTR^ and has been routinely used to study NGF-TrkA signaling. For our experiments, cells were starved of serum for 4 h in order to be synchronized prior to treatment with NGF (100 ng/mL) or ENT-A013 (500 nM) for 30′. Immunoprecipitation experiments with TrkA antibody and subsequent Western blot analysis against phosphorylated Tyrosine residues showed that compound ENT-A013 can indeed induce phosphorylation of TrkA (% of control FC 2.05 ± 0.21) at levels comparable to NGF (% of control FC 2.30 ± 0.62) (Figure 3A,B). In addition to that, this interaction is specific for TrkA, since ENT-A013 did not induce phosphorylation of other neurotrophin receptors TrkB or TrkC (Figure S2). It is especially notable that ENT-A013 does not activate the pro-apoptotic p75^NTR^ receptor. However, interestingly, it appears to partially inhibit this death receptor, albeit not statistically significantly (Supplementary Figure S1), an effect that could contribute to the compound’s neuroprotective role. Furthermore, ENT-A013 can also induce phosphorylation of Akt (% of control FC 3.8 ± 1.15) and Erk1/2 (% of control FC 2.5 ± 0.6), two kinases that are downstream of TrkA signaling and have been associated with NGF promotion of cell survival.

### 3.6. Both ENT-A013 Geometrical Isomers Activate TrkA Receptor and Its Downstream Signaling Kinase Erk1/2

Based on ENT-A013 structure, it is evident that it can be distinguished in two geometrical isomers, termed ENT-A013*E* and ENT-A013*Z*. In order to elucidate if there are any differences in the activity of each isomer on TrkA receptor activation, we treated PC12 cells with each isomer (500 nM) or NGF (100 ng/mL) for 30′ followed by immunoprecipitation with TrkA and subsequent Western blot analysis against Phosphorylated Tyrosine residues. Figure 3C,D shows that each of the isomers can indeed induce TrkA phosphorylation at similar levels with NGF treatment (% of control FC 59.4 ± 37.6 for ENT-A013E, 69.3 ± 46.14 for ENT-A013Z). Additionally, both isomers can activate downstream signaling kinase Erk1/2 (% of control FC 7.87 ± 2.23 for ENT-A013E, 4.13 ± 1.46 for ENT-A013Z), hence suggesting that both ENT-A013E and ENT-A013Z achieve NGF-mimicking action equally with regards to the ENT-A013 [*E,Z* (1:1) mixture of isomers].

### 3.7. ENT-A013, ENT-A013E and ENT-A013Z Sustain TrkA and Erk1/2 Phosphorylation Long Term and Up to 1 h after Treatment

We then investigated whether the ENT-A013-mediated activation of TrkA is a fast transient effect or can be sustained over longer periods of time. To this end, we treated PC12 cells with either compound (ENT-A013, ENT-A013E and ENT-A013Z) or NGF for 10, 30 or 60′. Figure 3E shows that indeed all three compounds can sustain TrkA phosphorylation for up to 1hr following treatment at comparable levels to NGF treatment, suggesting a different pattern of TrkA activation compared to the BNN27 compound, which shows different TrkA-Erk1/2 activity [17].

### 3.8. ENT-A013 Protects PC12 Cells from Serum Deprivation-Induced Cell Death through TrkA Receptor

**Figure 4:**
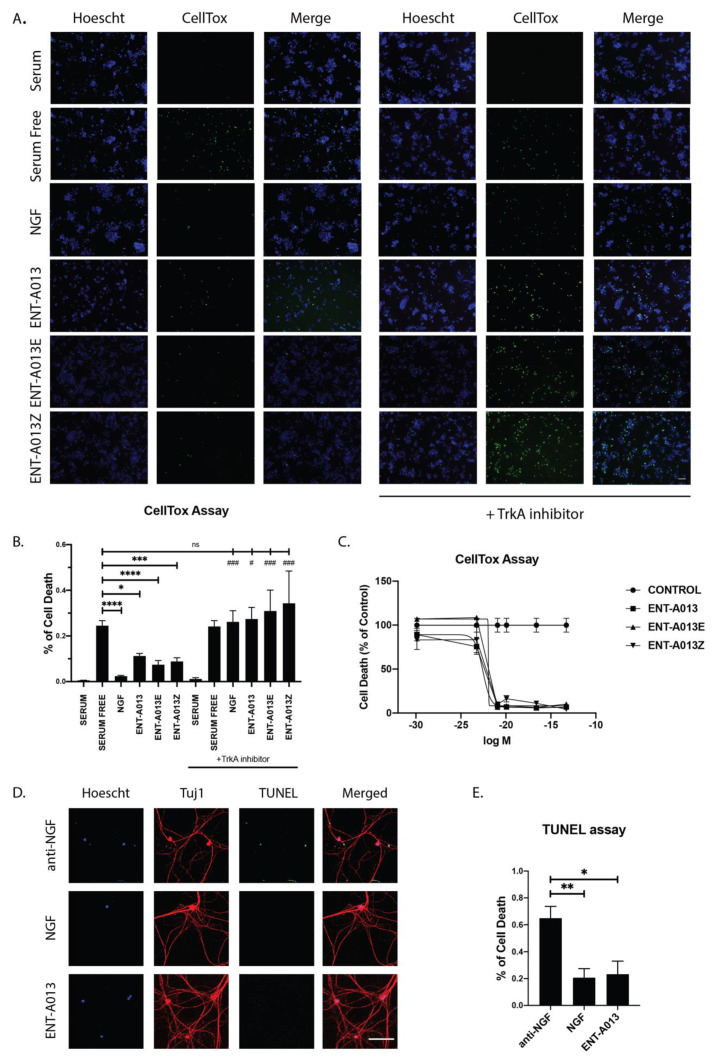
ENT-A013, ENT-A013E and ENT-A013Z protect PC12 cells against serum deprivation-induced cell death in a TrkA-dependent manner. PC12 cells were starved of serum and treated with NGF, ENT-A013, ENT-A013E, ENT-A013Z or vehicle in the presence or absence of a specific TrkA inhibitor GW441756 for 24 h and CellTox assay was used to quantify cell death. CellTox positive cell number was normalized to total Hoescht positive cell number in each image. Representative images from 4–9 independent experiments are shown (**A**) and quantification (**B**). Data represent ±SEM. One-way ANOVA, Tukey’s test correction: ns: non-significant; * and # *p* < 0.05; ** and ## *p* < 0.01; *** and ### *p* < 0.001; **** *p* < 0.0001. (**C**) Dose–response curve. IC_50_ value of compounds ENT-A013, ENT-A013E and ENT-A013Z calculated at 166.72 nM, 281.19 nM and 272.89 nM, respectively. (**D**,**E**) Primary DRG neurons were treated for 48 h with ENT-A013 in the absence of NGF and in the presence of an anti-NGF neutralizing antibody. TUNEL assay was used to assess neuronal cell death. Representative images from 3–5 independent experiments. Data are shown as ± SEM. One-way ANOVA, Tukey’s Test correction: * *p* < 0.05; ** *p* < 0.01.

Having established that compound ENT-A013 [*E,Z* (1:1) mixture] and its pure isomers ENT-A013E and ENT-A013Z induce sustained phosphorylation of TrkA and its downstream kinases, we then proceeded to investigate the potential anti-apoptotic effect of these compounds. We used PC12 cells treated for 24 h with our compounds or NGF in the absence of serum. Following treatment, cells were stained with CellTox reagent and Hoescht to identify apoptotic cells. Our compounds could protect cells from serum deprivation-induced cell death (0.11 ± 0.01 for ENT-A013, 0.07 ± 0.02 for ENT-A013E and 0.08 ± 0.02 for ENT-A013Z vs. 0.24 ± 0.02 for serum-deprived cells). Furthermore, we show that this anti-apoptotic effect is dependent upon the TrkA receptor’s activity since analysis of cells treated for 24 h with the compounds in the presence of a specific TrkA inhibitor revealed no protective effect (Figure 4A,B). Lastly, we performed a dose–response experiment to identify the IC_50_ and the optimal effective concentration of these compounds. All compounds showed strong anti-apoptotic effect at 500nM and up to 100 μM while no toxicity effects were observed. The IC_50_ for ENT-A013, ENT-A013E and ENT-A013Z was calculated at 166.72 nM, 281.19 nM and 272.89 nM, respectively (Figure 4C).

### 3.9. ENT-A013 Promotes Dorsal Root Ganglia Neuron Survival in the Absence of NGF

Following our results for the PC12 cell line showing that ENT-A013 can induce TrkA, Akt and Erk1/2 phosphorylation/activation, as well promoting cell survival in a TrkA-dependent manner, we then investigated whether this compound can have similar effects in a primary neuronal population whose survival and growth is highly dependent on NGF-TrkA signaling. Hence, we isolated Dorsal Root Ganglia neurons from P0-P1 mouse pups and cultured them for two weeks in the presence of NGF. At 14 days *in vitro* (DIV), the neuronal medium was replaced with one lacking NGF and supplemented with ENT-A013 and an NGF-neutralizing antibody in order to block the effect from endogenously produced and secreted NGF. Cells were treated for 48 h, then fixed, and cell death was assessed using TUNEL assay. ENT-A013 was able to significantly reverse the NGF deprivation-induced apoptosis, and notably, in similar levels with the NGF-treated neurons, indicating that this compound could effectively mimic NGF protective effects in neuronal populations that are dependent on the neurotrophin’s presence (Figure 4D,E). Additionally, we examined if ENT-A013 could promote axonal growth in the same neuronal population in the absence of NGF. At the moment of plating, cells were deprived of NGF and supplemented with ENT-A013 and an NGF-neutralizing antibody and cultured for 5 DIV. Cells were then fixed, stained with Tuj-1 antibody and the axonal length was measured. ENT-A013-treated cells showed a trend of increased axonal length; however, this difference was not significant compared to the NGF-deprived group (Figure S3). Collectively, this set of experiments showed that ENT-A013 is a potent and specific activator of TrkA and can promote cell survival not only in cell lines but also in primary neuronal populations, replacing NGF neuroprotective activity but not its axonal growth potential.

### 3.10. ENT-A013 Positively Modulates APP Processing and Protects Hippocampal Neurons from Aβ Toxicity

**Figure 5:**
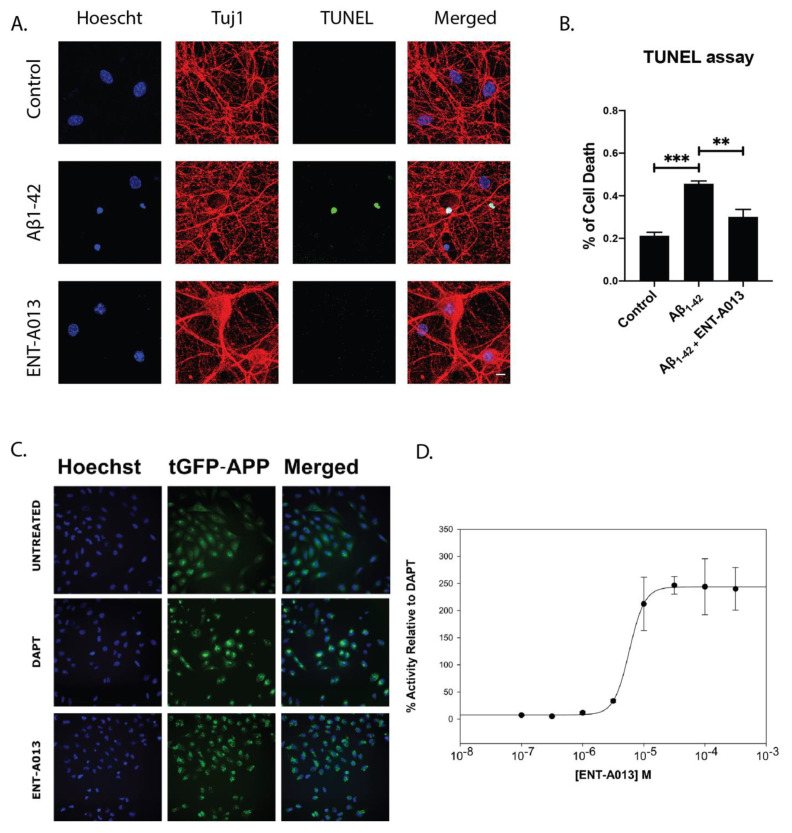
ENT-A013 shows anti-amyloid activity. (**A**,**B**) Primary hippocampal neurons were treated for 48 h with oligomeric Aβ in the presence of ENT-A013 and TUNEL assay was used to quantify cell death. Representative images from 3 independent experiments. Data are shown as ±SEM. One-way ANOVA, Tukey’s Test correction: ** *p* < 0.01; *** *p* < 0.001. (**C**) Aggregate formation on untreated cells, treated with DAPT inhibitor of γ-secretase or with compound ENT-A013. (**D**) IC_50_ value for ENT-A013 calculated at 92.950 μM and determined by treating U2OS APP-C99-tGFP cells with concentrations from 100 nM to 300 μM for 6 h. Aggregate formation was quantified with a BD Pathway 855 High-Content Bioimager and Attonvision software.

The Alzheimer’s Disease (AD) phenotype is partly attributed to the toxic effects caused by oligmeric Aβ, which results in reduced neuronal trophic support, caused by deregulation of neurotrophic signaling [2]. It is therefore reasonable that a small molecule that can selectively activate TrkA signaling and promote neuronal survival could alleviate the detrimental effects caused by toxic Aβ oligomers, especially during the early phases of the disease or modulate APP misprocessing and therefore reduce the production of the toxic Aβ. The U2OS stably expressing APP-C99-tGFP cell line was used in order to monitor γ-secretase activity, the process responsible for Aβ production from APP. The assay achieves this by measuring the levels of fluorescent APP-C99 construct produced by β-secretase activity. When γ-secretase is active, the construct is cleaved, leading to Aβ production. In the presence of a γ-secretase inhibitor, this processing does not occur, and the construct accumulates in the plasma membrane causing an increase in fluorescence. Therefore, higher fluorescence levels correspond to decreased γ-secretase activity and Aβ aggregate formation by extension. Fluorescence levels increased after treatment with γ-secretase inhibitor DAPT at 3 μM, used as a positive control. Treatment for 6 h with the compound ENTA-013 also showed an increase in fluorescence (decrease of γ-secretase activity) in a dose-dependent manner, at increasing concentrations (100 nM to 300 μM). The activity of the compound ENTA-013 in reducing γ-secretase activity, compared to untreated cells was quantified relative to that of DAPT, as shown in Fig. 5C ENTA-013 concentrations of 10 μM, 30 μM, 100 μM and 300 μM lead to increases in APP spot levels of 166.3%, 246.4%, 243.9% and 239.9%, respectively, compared to DAPT, while the IC50 value for ENTA-013 is 92.590 μM. In addition, in order to investigate whether ENT-A013 can protect cells against Aβ-induced toxicity we isolated neurons from the hippocampus of E17.5 mouse embryos, a brain area that is heavily affected in AD. Neurons were left in culture for 16–18 DIV and then were treated with oligomeric Aβ and ENT-A013 for 48 h. Subsequently, cells were fixed and cell death was assessed using TUNEL assay. ENT-A013-treated cells showed significantly decreased apoptosis (29.8% ± 2.5%) compared to Aβ-treated ones (41.5% ± 4.2%) and similar to control cells (18.7% ± 2.8%) (Figure 5A,B). Interestingly, ENT-A013 treatment also reduced phosphorylated JNK levels similar to untreated control cells (Figure S4), indicating the inhibition of cJun pro-apoptotic pathway. These results suggest that ENT-A013 can protect primary hippocampal neurons from oligomeric Aβ-induced cell death, introducing a significant therapeutic potential of this molecule against a major pathological factor of AD.

### 3.11. ENT-A013 Protects Hippocampal Synapses from Aβ-Induced Synapse Degeneration

**Figure 6:**
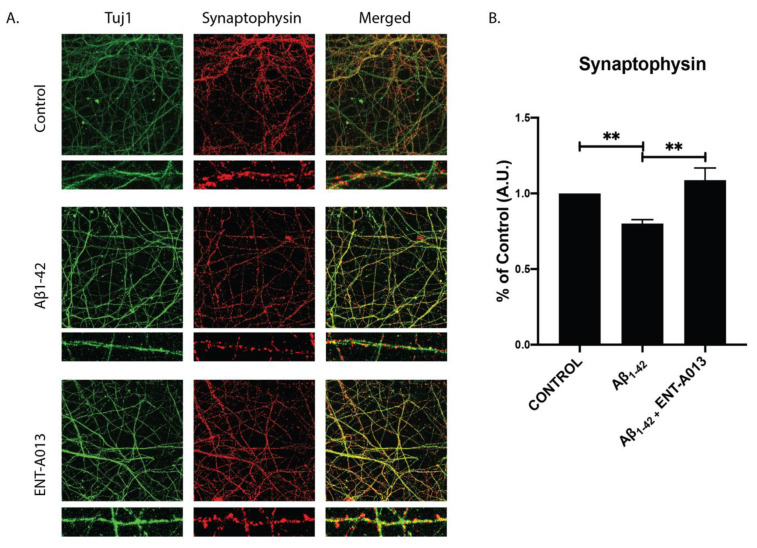
ENT-A013 protects against Aβ-induced synapse loss. (**A**,**B**) Primary hippocampal neurons treated for 4 h with oligomeric Aβ in the presence of ENT-A013 and stained against synaptophysin to assess synapse number. Total Synaptophysin area was normalized to total Tuj1 area in each image. Representative images from 4–5 independent experiments. Data are shown as ±SEM. One-way ANOVA, Tukey’s Test correction: ** *p* < 0.01.

Synapse degeneration is a major hallmark of AD, occurring early in the disease’s progression, and has been strongly correlated with deficiencies in learning and memory among AD patients [63,64]. Additionally, it has been shown that neurotrophin signaling plays a crucial role in regulating synapse number as well as enhancing synaptic strength and plasticity [65]. In order to investigate whether our compound can protect against Aβ-induced synapse degeneration we used primary hippocampal neurons treated with Aβ and ENT-A013. Cells at 14–16 DIV were treated for 4 h, and the synapse number was assessed using immunostaining against synaptophysin, a pre-synaptic marker. Figure 6 shows that the ENT-A013-treated group synapse number higher (% of control FC 1.08 ± 0.08) compared to the Aβ-treated one (% of control FC 0.78 ± 0.03). Collectively, our results suggest that ENT-A013 can ameliorate several aspects of Aβ-induced pathology in primary neuronal cells.

### 3.12. ENT-A013 Partially Reverses Long-Term Potentiation (LTP) in Brain Slices Treated with Oligomeric Aβ

**Figure 7:**
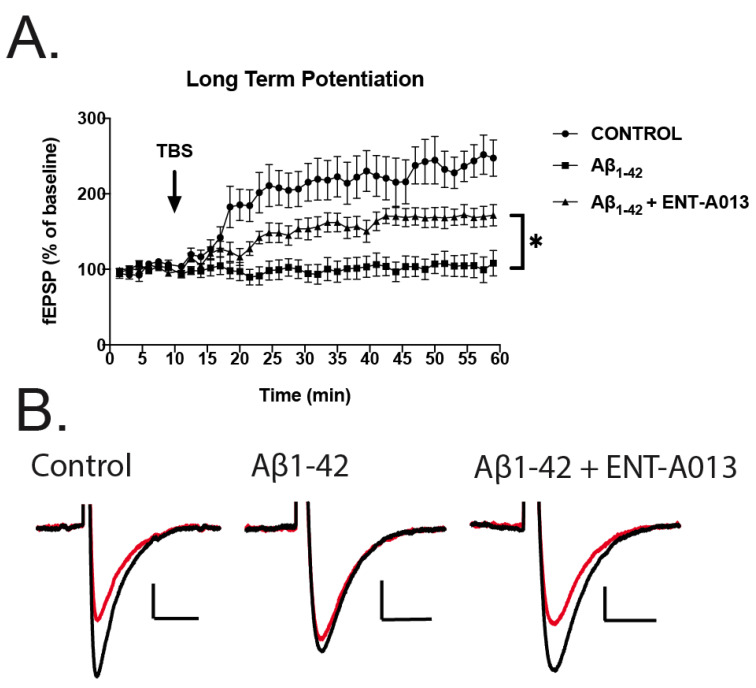
LTP in hippocampal brain slices treated with oligomeric Aβ (5 μM) in the presence or absence of ENT-A013 (500 nM) for 3–5 h. (**A**) Graph showing LTP induced with TBS in control brain slices (i.e., no treatment, circles), in brain slices treated with oligomeric Aβ (5 μM) and in brain slices treated with oligomeric Aβ (5 μM) (squares) and ENT-A013 (500 nM) (triangles). Statistical analysis ANOVA shows that ENT-A013 treated brain slices have significantly higher synaptic potentiation compared to those treated only with Aβ. Error bars represent SEM. Mixed effects multiple comparison analysis. N = 7–9 slices from 3–4 animals. * *p* < 0.05 (**B**) Representative traces before (red) and after (black) theta-burst stimulation for the 3 different groups.

We next sought to investigate whether ENT-A013 can ameliorate the toxic effects of Aβ in impairing LTP in brain slices from wild type animals. Impaired LTP is an important feature of AD and is considered to be a cellular mechanism that underlies memory and learning deficits, especially in the hippocampal region [66]. Brains from wild type animals were sliced transversely and treated with oligomeric Aβ (5 μM) in the presence or absence of ENT-A013 (500 nM) for 3–5 h and then subjected to LTP protocol. Field Excitatory Postsynaptic Potentials (fEPSPs) were recorded. Figure 7 shows that control brain slices exhibited significant synaptic potentiation following Theta-Burst Stimulation (TBS). More importantly, TBS in brain slices treated with Aβ and ENT-A013 exhibited significantly increased synaptic potentiation compared to brain slices treated only with Aβ, therefore further supporting the notion that ENT-A013 can be an effective drug in counteracting the deleterious effects of Aβ in synaptic functioning, and potentially restoring cognitive performance in AD patients.

## 4. Discussion

In the present study, we provide experimental evidence that ENT-A013 is a selective TrkA agonist, which mimics NGF activity in cell lines and primary neuronal populations and exhibits neuroprotective and anti-amyloid properties. Compared to our previously published work describing other related DHEA-derivatives BNN27 and BNN20, ENT-A013 [*E,Z* (1:1) mixture] and its pure geometrical isomers [ENT-A013E and ENT-A013Z] show selectivity and stronger specificity to the TrkA receptor. Indeed, ENT-A013, ENT-A013E and ENT-A013Z can strongly phosphorylate the TrkA receptor but not TrkB, TrkC or p75^NTR^. Interestingly, molecular docking studies revealed two possible binding sites for ENT-A013E and ENT-A013Z on the TrkA-NGF complex, sites 1a and 1b, with the latter providing a more stable interaction between the compound and the receptor, similar to that proposed for BNN27. Although these two similar compounds may interact similarly with the receptor, they have somewhat different activities. Furthermore, isozyme-specific CYP450 experiments revealed that ENT-A013 is only a weak-to-moderate inhibitor of some CYP450 drug-metabolizing isoforms, and does not show biodegradation or liver metabolism, as is the case for BNN27 (70% residual of time zero at t = 60 min in human cryopreserved hepatocytes, also supported by mouse pharmacokinetic data) or safety issues [67,68]. It therefore appears to be a well-tolerated, druggable compound.

We show that ENT-A013, ENT-A013E and ENT-A013Z can induce sustained phosphorylation of TrkA and Erk1/2 for up to 1hr after treatment, while BNN27 can phosphorylate TrkA for up to 6hr but fails to activate Erk1/2 past 15 min. Additionally, ENT-A013 ENT-A013E and ENT-A013Z were effective in providing protection against serum deprivation and NGF withdrawal-induced apoptosis in PC12 cells and primary DRG neurons, respectively, and therefore mimic the neuroprotective properties of NGF, though they failed to induce neurite outgrowth. These differential NGF effects by ENT-A013 as an equimolar mixture of the two geometrical isomers or as the pure ENT-A013E and ENT-A013Z could reflect structural-induced differences in various signaling pathways and cellular phenotypes between the two molecules, thus providing us the opportunity to decipher the signaling pathways that mediate preferable vs. non-preferable effects of the neurotrophin, for instance neuroprotection vs. hyperalgesia. ENT-A013 actions are mediated through NGF-TrkA signaling, since pharmacological inhibition of TrkA with a specific inhibitor completely abolishes them.

Moreover, ENT-A013 could successfully diminish harmful Aβ-induced effects such as neuron loss and synapse degeneration. Experiments in primary hippocampal neurons revealed that ENT-A013 had a strong effect in reversing both neuronal cell death and synapse loss, two cellular processes that are highly affected in AD, thus indicating a pleiotropic mechanism of action for this molecule. Importantly, apart from synapse number, ENT-A013 was also able to restore synaptic potentiation in brain slices treated with Aβ, a cellular process that is heavily affected in AD patients and is strongly correlated with cognitive decline. Lastly, we showed that ENT-A013 could modulate APP processing shifting towards the non-amyloidogenic pathway, similarly to DHEA [69,70], and thus provide a mechanism of action for this compound against the toxic Aβ effects.

NGF signaling is crucially important in the development, growth and functioning of neurons in the central and peripheral nervous system [1,71]. Indeed, in AD mouse models and human patients there is strong association between the deregulation of NGF signaling and the appearance of major disease hallmarks. The NGF-TrkA receptor complex of the basal forebrain cholinergic neurons (BFCN), which is a population of neurons heavily affected early in the progression of AD, has been shown to be disturbed in multiple ways [72,73,74,75]. During the progress of AD, decreased NGF processing results in an increase in pro-NGF compared to mature NGF concomitantly with the loss of the TrkA receptor, which in turn shifts the balance towards increased pro-NGF/p75^NTR^ pro-apoptotic signaling [8,76]. Additionally, single-neuron gene expression profile and network analysis provided a strong association between hampered neurotrophin signaling and AD susceptibility [77]. These data support the merging of two old hypotheses for the emergence of AD, the cholinergic and neurotrophic ones [78,79]. It is the deregulation of NGF signaling that causes the BFCNs to collapse and this initiates the AD pathology. It is therefore intriguing that the external provision of NGF could reverse these effects and ameliorate AD pathology.

Presently, available drugs against AD target acetylcholinesterase and only offer modest symptomatic relief. It is therefore of the utmost importance to discover so-called “disease modifying” therapies or drugs [80,81,82]. The FDA has approved the first drug for AD, which targets Aβ peptide, alas sparking controversy among experts [83,84,85]. Several other attempts to target Aβ plaques or peptide have been recently declared unsuccessful [86,87,88] and many more are in late-stage clinical trials [89]. Similarly, delivery of NGF by means of intracerebroventricular injections or AAV2-NGF gene therapy has also been unsuccessful due either to undesired effects or a lack of improvement for patients [9,11,12]. In contrast, a small molecule that is BBB-permeable, has good pharmacokinetic properties and can act as an NGF-mimetic is a good candidate to counteract the harmful Aβ-induced phenotype presented in AD, with important therapeutic potential [90,91].

To conclude our findings, we have described ENT-A013, a novel, small-sized molecule, which is a potent and selective TrkA agonist, showing strong neuroprotective properties, protecting hippocampal neurons against Aβ toxicity. ENT-A013 constitutes a novel lead molecule that could serve as a prototype TrkA agonist for further preclinical development in AD animal models and provide a new category of therapeutic agents in the fight against AD by enhancing the neuronal survival of the affected cell populations.

## Data Availability

All materials are available upon request. Synthetic compounds are available under a Material Transfer Agreement with the University of Crete, FORTH and National Hellenic Research Foundation.

## Supplementary Materials

The following are available online at https://www.mdpi.com/article/10.3390/biomedicines10030614/s1: Supporting Information S1: Synthesis of ENT-A013; Figure S1: ENT-A013 does not activate p75^NTR^; Figure S2: ENT-A013 does not activate TrkB nor TrkC; Figure S3: ENT-A013 does not promote neurite length in primary DRG neurons in the absence of NGF; Figure S4: ENT-A013 protects hippocampal neurons against amyloid toxicity by inhibiting JNK pathway; Table S1: List of antibodies used in this study; Table S2: Excitation and emission wavelengths for the CYP450-specific fluorescent substrates.
