# Supplementary material for: Development and Biological Characterization of a Novel Selective TrkA Agonist with Neuroprotective Properties against Amyloid Toxicity
Source: Biomedicines. 2022 Mar 6;10(3):614. doi: 10.3390/biomedicines10030614 (PMC8945229; doi:10.3390/biomedicines10030614)
Supplement: Supplementary file 1 [file biomedicines-10-00614-s001.zip › biomedicines-1573970-supplementary.pdf]

Antibody	Concentration	Manufacturer	Catalog number	Application
TrkA	1:100/1:1000	Sigma-Aldrich	06-574	IP/WB
p75	1:100	Abcam	ab6172	IP
Phosphorylated Tyrosine	1:1000	R&D systems	BAM1676	WB
Traf6	1:2000	Abcam	Ab33915	WB
Phosphorylated TrkB	1:1000	Sigma-Aldrich	ABN1381	WB
phosphorylated TrkC	1:1000	St John's Laboratory	STJ90960	WB
TrkB	1:1000	Sigma-Aldrich	07-225-I	WB
TrkC	1:1000	Cell Signalling	C44H5	WB
p75	1:1000	Biolegend	839701	WB
phosphorylated Akt	1:1000	Cell Signalling	9721S	WB
phosphorylated Erk1/2	1:1000	Cell Signalling	9101S	WB
Akt	1:1000	Cell Signalling	4691S	WB
Erk1/2	1:1000	Cell Signalling	4695S	WB
NGF	1:500	Sigma-Aldrich	N8773	Neutralization
Tuj1	1:2000	Biolegend	801201	IF
Synaptophysin	1:1000	Invitrogen	PA1-1043	IF
Phosphorylated JNK	1:1000	Cell Signalling	4668S	WB
JNK	1:1000	Cell Signalling	9252S	WB
anti-mouse Alexa fluor 488	1:1000	Invitrogen	A-11029	IF
anti-rabbit Alexa fluor 546	1:1000	Invitrogen	A10040	IF
anti-mouse Cy3	1:1000	Invitrogen	A10521	IF

Supplementary Table S1: List of antibodies used in this study

CYP450 isoforms	Conc [μM]	Fluorescent signal	Excitation (nm)		Emission (nm)	
			center	band width	center	band width
CYP1A2	3	Blue	415	20	460	20
CYP2B6	3	Blue	415	20	460	20
CYP2C9	1	Red	550 (535)	12 (25)	590 (20)	12
CYP2C19	10	Blue	415	20	460	20
CYP2D6	10	Blue	415	20	460	20
CYP3A4	3	Red	550	12	590	12

Supplementary Table S2. Excitation and emission wavelengths for the CYP450-specific fluorescent substrates

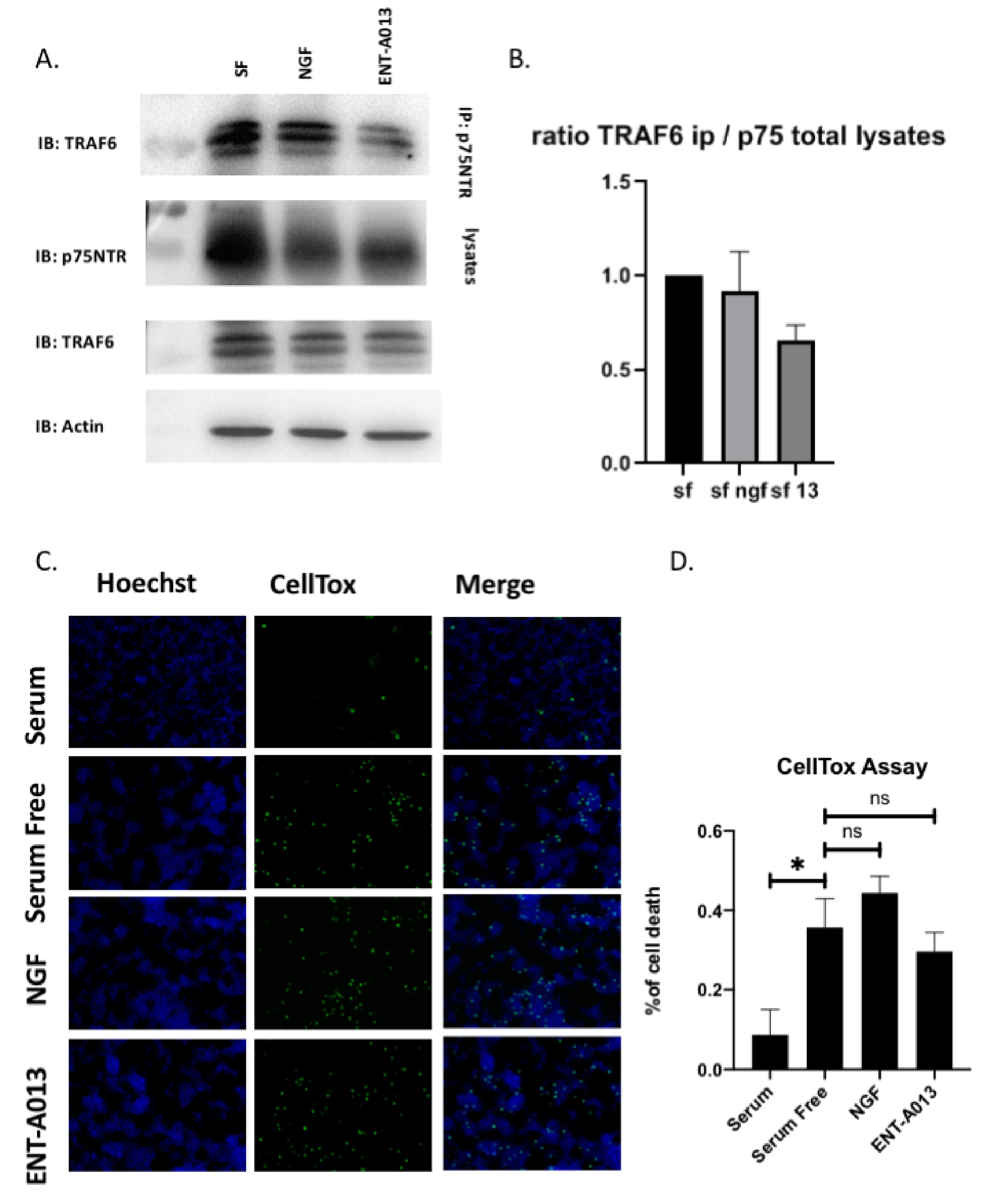

Supplementary Figure S1: ENT-A013 does not activate p75^{NTR}. (A) HEK cells transfected with p75^{NTR} and Traf6 were treated with ENT-A013 (500nM) or NGF (100ng/ml) for 30' and then were subjected to immunoprecipitation experiments against p75. Quantification (B) shows that ENT-A013 does not activate p75^{NTR}. (C) HEK cells transfected with p75^{NTR} were starved from serum and treated with ENT-A013 (500nM) or NGF (100ng/ml) for 24hrs and subsequently subjected to CellTox assay. Quantification (D) shows that there is no significant difference between ENT-A013 treated group and negative control (Serum Free).

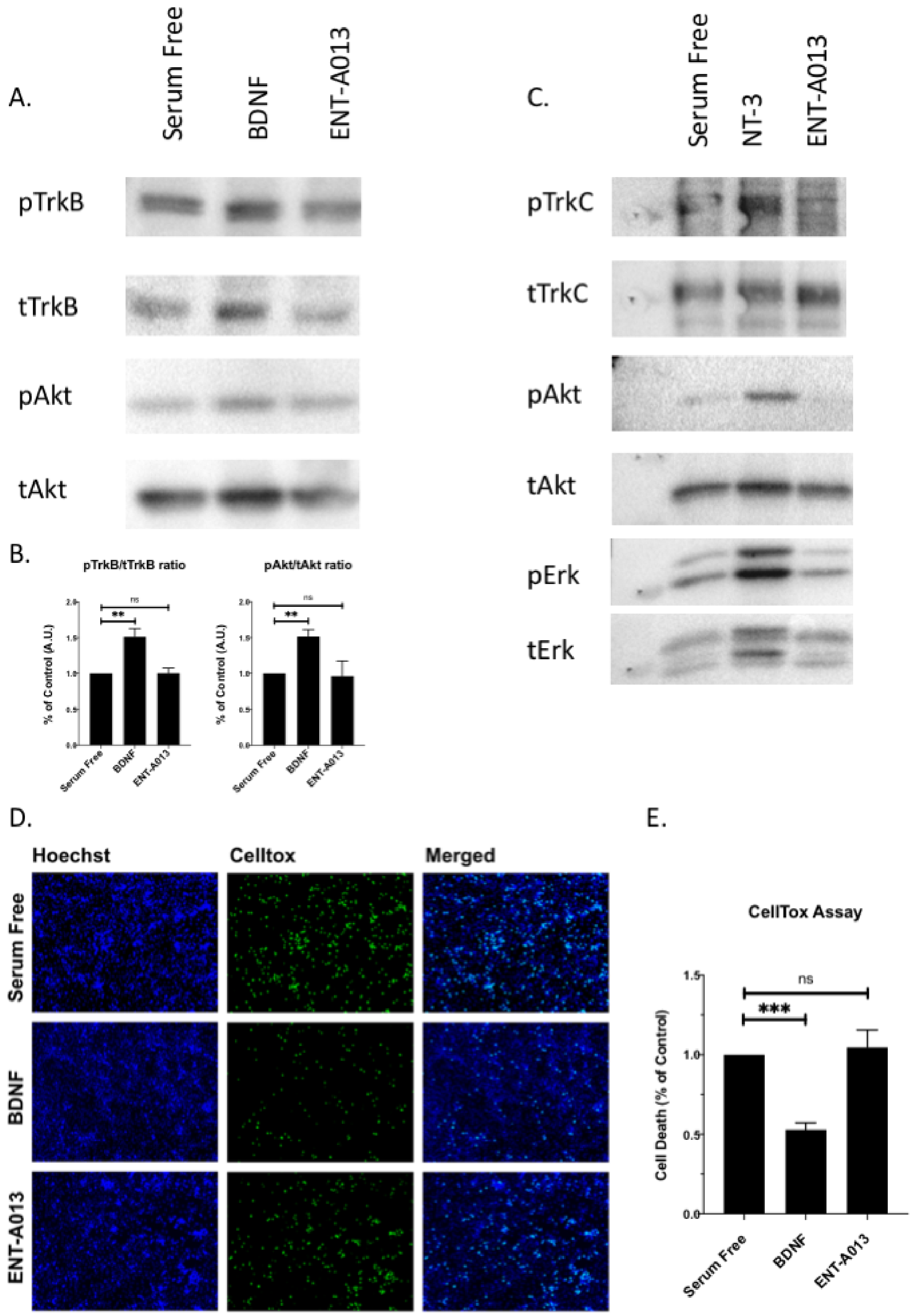

Supplementary Figure S2: ENT-A013 does not phosphorylate TrkB or TrkC. (A, B) NIH-3T3 cells were stably transfected with TrkB and treated with ENT-A013 (500nM) or BDNF (500ng/ml) for 30' and then subjected to Western Blot against phosphorylated TrkB and Akt. Quantification shows that there is no significant difference in the phosphorylation of either TrkB or Akt by ENT-A013. (C) NIH-3T3 cells were stably transfected with TrkC and

treated with ENT-A013 (500nM) or Neurotrophin-3 (100ng/ml) for 30' and then subjected to Western Blot against phosphorylated TrkC, Akt and Erk1/2. ENT-A013 failed to phosphorylated any of those. (D) CellTox assay was performed in NIH-3T3-TrkB cells that were starved from serum and treated with ENT-A013 or BDNF for 24hrs. Quantification (E) shows that there is no significant difference between the ENT-A013 treated group and negative control (Serum Free).

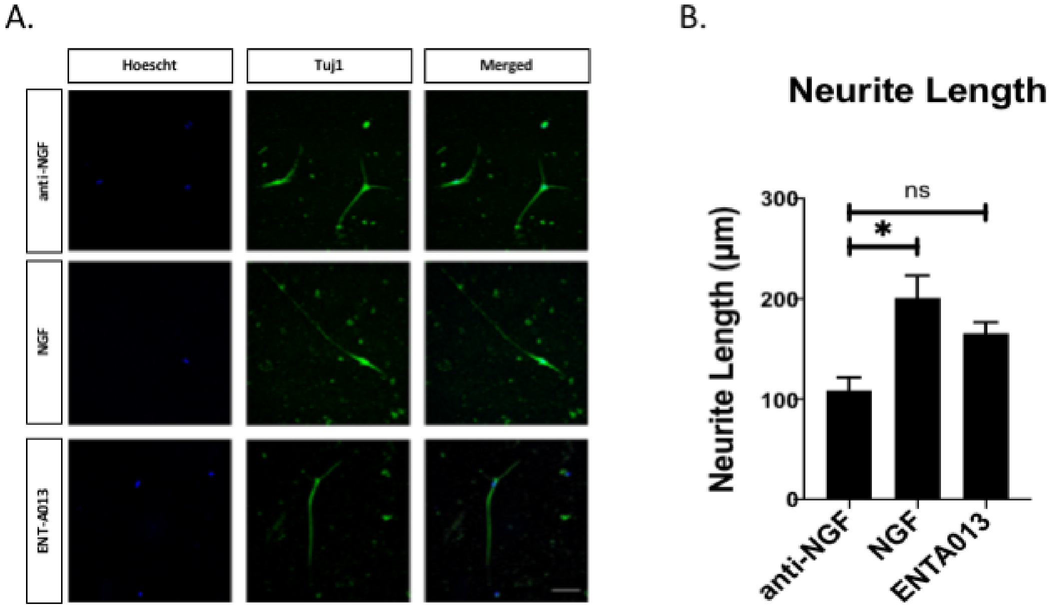

Supplementary Figure S3: Primary DRG neurons were cultured in the presence of ENT-A013 (500nM, supplemented every 48hrs) and a neutralizing antibody against NGF for 5 days and then neurite length was measured by immunostaining against Tuj1. ENT-A013 failed to significantly increase neurite outgrowth in primary DRG neurons in the absence of NGF.

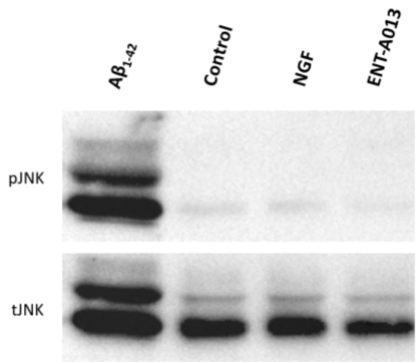

Supplementary Figure S4: Primary hippocampal neurons at 14-16 DIV were treated with oligomeric Aβ and in the presence of ENT-A013 (500nM, supplemented every 24hrs) for 48hrs and then were subjected to Western Blot against phosphorylated JNK. A representative blot from n=2 experiments shows that ENT-A013 was able to prevent JNK phosphorylation.
